# Predictors of liver disease progression in people living with HIV-HBV co-infection on antiretroviral therapy

**DOI:** 10.1016/j.ebiom.2024.105054

**Published:** 2024-03-21

**Authors:** Kasha P. Singh, Anchalee Avihingsanon, Jennifer M. Zerbato, Wei Zhao, Sabine Braat, Surekha Tennakoon, Ajantha Rhodes, Gail V. Matthews, Christopher K. Fairley, Joe Sasadeusz, Megan Crane, Jennifer Audsley, Sharon R. Lewin

**Affiliations:** a Department of Infectious Diseases, The University of Melbourne at the Peter Doherty Institute for Infection and Immunity, Melbourne, Victoria, 3000, Australia; b Victorian Infectious Diseases Service, Royal Melbourne Hospital at the Peter Doherty Institute for Infection and Immunity, Melbourne, Victoria, 3000, Australia; c Department of Infectious Diseases, Alfred Health and Monash University, Melbourne, Victoria, 3004, Australia; d HIV-NAT, Thai Red Cross AIDS Research Center (TRCARC), Bangkok, 10330, Thailand; e Centre for Epidemiology and Biostatistics, School of Population and Global Health, The University of Melbourne, Melbourne, Victoria, 3053, Australia; f MISCH (Methods and Implementation Support for Clinical Health) research Hub, Faculty of Medicine, Dentistry and Health Sciences, The University of Melbourne, Melbourne, Australia; g Kirby Institute, UNSW, Kensington, New South Wales, 2052, Australia; h Melbourne Sexual Health Centre, Alfred Health, Carlton, 3053, Australia

**Keywords:** HIV, Hepatitis, Liver, Fibrosis, HMGB1

## Abstract

**Background:**

In people living with HIV-HBV, liver fibrosis progression can occur even with suppressive antiretroviral therapy (ART). We investigated the relationship between liver fibrosis and biomarkers of inflammation, apoptosis, and microbial translocation.

**Methods:**

In this observational cohort study adults living with HIV-HBV already on effective ART were recruited in Australia and Thailand and followed for 3 years including 6 monthly clinical review and blood tests and annual transient elastography. Differences in clinical and laboratory predictors of liver fibrosis progression were tested followed by regression analysis adjusted for CD4+ T-cells at study entry. A linear mixed model was fitted to longitudinal data to explore changes over time.

**Findings:**

67 participants (85% male, median age 49 y) were followed for 175 person-years. Median duration of ART was 10 years (interquartile range (IQR) 8–16 years). We found 11/59 (19%) participants during 3-years follow-up (6/100 person-years) met the primary endpoint of liver disease progression, defined as increased Metavir stage from baseline to final scan. In regression analysis, progressors compared to non-progressors had higher levels of high mobility group box 1 protein (HGMB1), (median (IQR) 3.7 (2.6–5.0) and 2.4 ng/mL (1.5–3.4) respectively, adjusted relative risk 1.47, 95% CI [1.00, 2.17]) and lower nadir CD4+ T-cell percentage (median 4% (IQR 2–8) and 11% (4–15) respectively (relative risk 0.93, 95% CI [0.88, 0.98]).

**Interpretation:**

Progression in liver fibrosis occurs in people with HIV-HBV on suppressive ART. Fibrosis progression was associated with higher HMGB1 and lower percentage nadir CD4+ T-cell count, highlighting the importance of early initiation of HBV-active ART.

**Funding:**

This work was supported by 10.13039/501100000925NHMRC project grant 1101836; 10.13039/501100000925NHMRC practitioner fellowship 1138581 and 10.13039/501100000925NHMRC program grant 1149990. The funder had no role in study design, data collection, data analysis, interpretation, writing of this manuscript or decision to submit for publication.

**Research in context**

**Evidence before this study**

People living with both HIV and HBV have higher rates of liver disease progression to fibrosis (scarring) than those living with HBV alone. Treatment with antiretroviral therapy that is active against both HIV and HBV has improved outcomes with reduced morbidity and mortality for individuals living with HIV and HBV. However, in contrast to those with HBV alone, in whom progression of liver fibrosis is uncommon on treatment (<1% after 5 years), previous studies have shown 8–25% of people have progression of liver fibrosis despite antiretroviral therapy that is active against and suppresses both viruses. The reason for this is unclear. Previous studies have found that liver fibrosis was associated with lower CD4+ T cells, some HBV genotypes or HBV viral load. Prior studies have been limited by being retrospective and having short duration of follow up and surrogate scoring systems for assessment of liver fibrosis.

**Added value of this study**

We prospectively followed people living with HIV and HBV on suppressive antiviral treatment over 3 years and measured liver fibrosis annually using transient elastography (Fibroscan®) and quantified markers of inflammation, immunity and microbial translocation in serum collected every 6 months. We showed that higher serum levels of high mobility group box 1 (HMGB1), a damage associated molecular protein which increases with cellular apoptosis, at the time of enrolment and lower CD4+ T cell nadir (%) was associated with liver fibrosis progression.

**Implications of all the available evidence**

This study supports the early initiation of antiretroviral therapy in people with HIV-HBV co-infection and that an increase in cell death is associated with liver fibrosis progression. The marker HMGB1 is not specific to the liver, however in a previous study, our group showed increased cell death in the liver and higher levels of serum HMGB1 in people with HIV-HBV compared to people with HBV alone. Together these data support the possibility of HMGB1 as a mediator of fibrogenesis in the liver, and the potential for HMGB1 to be investigated as a predictive biomarker to identify individuals who may be at risk for liver fibrosis progression.

## Introduction

The widespread availability of antiretroviral therapy (ART) active against both human immunodeficiency virus (HIV) and hepatitis B (HBV) has led to significant improvements in morbidity and mortality in people living with HIV (PWH) and HBV co-infection.^1^, ^2^, ^3^ Despite treatment, liver disease occurs at higher rates in PWH and HBV co-infection compared to PWH or with HBV alone.^4^, ^5^, ^6^ In people with HBV mono-infection, tenofovir treatment is associated with fibrosis regression in over 50% of people, as well as reversal of cirrhosis both on biopsy and as a reduction in liver stiffness measurement (LSM) by transient elastography (TE) (Fibroscan®).^7^^,^^8^ Fibrosis progression can occur but is estimated at <1% after 5 years.^7^^,^^8^ In contrast, in PWH and HBV co-infection, 8–25% of people are reported to have progression despite tenofovir containing ART.^5^^,^^9^, ^10^, ^11^, ^12^, ^13^, ^14^, ^15^, ^16^, ^17^, ^18^, ^19^ Understanding predictors and pathogenesis of liver fibrosis progression in PWH and HBV co-infection on ART will help improve management strategies.

Liver fibrosis progression in PWH and HBV co-infection on HBV-active ART has been associated with age,^9^^,^^13^^,^^15^^,^^19^ male sex,^5^^,^^13^^,^^15^ lower CD4+ T-cells,^9^^,^^13^^,^^19^ lower nadir CD4+ T-cells,^5^ longer ART duration,^19^ HBV genotype G,^9^ higher fasting glycemia or anemia^5^ and hepatic steatosis,^17^ whilst fibrosis regression has been associated with lower BMI,^9^^,^^14^ higher CD4+ T-cells^5^^,^^9^^,^^14^ higher HBV viral load (VL) at first TE,^11^ suppressed HIV VL^15^ and lower fasting triglycerides.^5^ Interpretation of these studies is complex, because some studies used surrogate scoring systems for fibrosis assessment including serum markers (e.g. ‘FIB-4’, ‘FibroTest’, AST to Platelet Ratio Index (APRI)), while others used LSM.

We investigated liver disease over 36 months in PWH and HBV co-infection already on suppressive ART in a prospective observational cohort to quantify rates of liver disease progression and to identify factors that may be associated with progression of fibrosis.

## Methods

#### Ethics

The study was approved by Human Research Ethics Committees (Melbourne–Alfred Health (76/12), Sydney-St Vincent's Hospital (12/SVH/235), Thailand-Faculty of Medicine, Chulalongkorn University Institutional Review Board (HIV-NAT 178.1) and was conducted in compliance with ethical approval. All participants provided informed consent to participate in the study.

#### Study participants

**Supplementary Figure S1:**
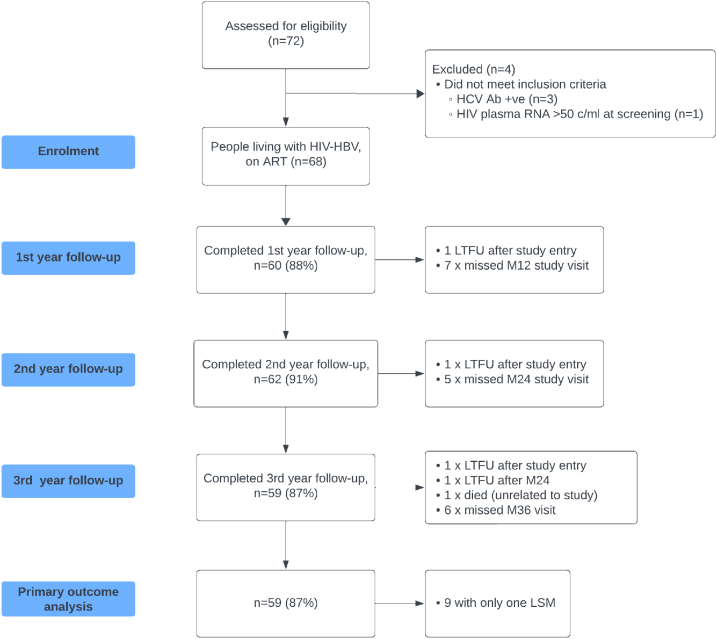
**Strobe Diagram of screening and study enrolment**.

Adults with HIV and HBV co-infection were recruited in Thailand and Australia. Recruitment was from outpatient clinics at the Alfred Hospital, Melbourne, Australia; Melbourne Sexual Health Centre, Melbourne, Australia; St Vincent's Hospital, Sydney, Australia; and the HIV-Netherlands-Australia-Thai Research Collaboration (HIV-NAT), Thai Red Cross AIDS Research Centre, Bangkok, Thailand. The recruitment period was from October 2012–February 2014 with final follow-up visits completed by February 2017. Those included had already received HBV-active ART for at least 12 months with viral suppression (HBV DNA<351 IU/mL, HIV RNA<50 copies/mL, due to assays in regular use) and negative HCV antibody (Ab) at screening (Supplementary Figure S1). Participants were followed prospectively for 3 years with 6-monthly visits and annual LSM.

Demographics and ART history were collected. At each visit blood samples were taken, and participants completed questionnaires on alcohol intake and medication adherence. ‘Alcohol excess’ was defined by the following responses. “How often have you had alcoholic drinks” answered as ‘daily/almost daily’, “number of standard drinks on a typical day when you are drinking” answered as ‘≥10’ OR “over past 6 months, how often have you had ≥ six drinks on one occasion” answered as ‘daily/almost daily’. Local laboratories completed standard of care testing (full blood count; liver function, CD4+/CD8+ T-cell counts, HIV RNA, HBV DNA, HBV serology (hepatitis B surface (HBs) and hepatitis B pre-core (HBe) antigen, anti-HBs and anti-HBe Ab levels).

#### Liver fibrosis

Liver fibrosis was assessed using LSM by TE by experienced operators within 12 months of study enrolment and at months 12, 24 and 36. We used TE cut-offs validated for PWH with HBV coinfection to stage fibrosis according to the Metavir system (F1–F4) (F1 < 5.9 kPa (kilopascals), F2 = 5.9–7.5 kPa, F3 = 7.6–9.3 kPa, F4 ≥ 9.4 kPa^20^^,^^21^).

##### Liver fibrosis progression

The pre-determined primary endpoint was a categorical change in liver fibrosis score, defined as an increase in Metavir fibrosis stage (F1 < 5.9 kPa (kiloPascals), F2 5.9–7.5 kPa, F3 7.6–9.3 kPa, F4 ≥ 9.4 kPa)^20^, ^21^ from baseline result to last study time point. These participants are referred to as “progressors”. Participants with fewer than two LSM were excluded. We used a pre-determined secondary definition of fibrosis progression of 20% increase and ≥2 kPa (at least one value >5.9 kPa).^22^ An exploratory analysis included defining ‘regressors’, based on a similar decrease in LSM.

#### Laboratory methods

##### Markers of microbial translocation, and immune mediators in plasma

These were measured at baseline, months 12, 24 and 36. Plasma soluble CD14 (sCD14) is a marker of microbial translocation and was measured as previously described^23^ and HMGB1, a marker of cell damage and death quantified by ELISA according to the manufacturer's instructions (Catalogue number 30164033 IBL International GMBH, Hamburg, Germany).^24^ Immune mediators including tumour necrosis factor (TNF)-α, interleukin (IL)-10 and IL-18, chemokine (C-X-C motif) ligand (CXCL)−9, −10, −11, C–C motif chemokine ligand (CCL)2, CCL3, CCL4 and CCL5 were measured by multiplex (Custom-LX, Luminex, Procartaplex, ThermoFisher Scientific, Waltham MA).

#### Sample size

No sample size calculation was done for this sub-study to assess predictors of liver disease progression. Participants were recruited as part of a larger multi-centre cross sectional and longitudinal cohort which had multiple aims related to liver disease pathogenesis in HIV-HBV co-infection^25^ and HIV persistence in the liver.^26^ As part of this exploratory analysis, effect sizes and confidence intervals in addition to p-values are reported to support interpretation of the results.

#### Statistical analysis

Baseline data including patient characteristics and plasma biomarkers at study entry were summarised using frequency and percentage (categorical data) and mean and standard deviation or median and interquartile range (IQR) (25th–75th percentile) (numerical data) depending on the symmetry of the distribution in total, by country (Australia or Thailand), and by progression status (progressor or non-progressor). Differences between progressors and non-progressors were tested using the Welch's t-test or Wilcoxon rank-sum test if non-normally distributed (numerical data) and chi-square test or Fisher's exact test as appropriate based on expected cell count (categorical data). In addition, progressors and non-progressors were compared using logistic regression to account for differences in CD4+ T-cells at study entry (except for variables related to CD4) and presented alongside adjusted relative risk estimates and corresponding two-sided 95% confidence intervals obtained using STATA command ADJRR.

A linear mixed regression model with random intercept for individual and random slope for time and unstructured covariance matrix between the random effects was fitted using restricted maximum likelihood to the longitudinal data of each clinical and biochemical marker separately. To examine the average 12-monthly rate of change we assumed a linear trajectory from baseline to month 36 (i.e., continuous time). The models were adjusted for CD4+ T-cells at study entry (except for the outcome variables measuring CD4+ T-cell count and CD4+ T-cells [%]). In addition, we compared the average 12-monthly rate of change between progressors and non-progressors by including the main effect for progressor status and its interaction with time in the model. Clinical and biochemical characteristics were log base e transformed before fitting models (except albumin). Before log-transformation, zero values for CCL2, CCL3, CCL4, IL-10 and IL-18, were replaced by the lower limit of detection divided by 2.

Pre-specified subgroups were explored for LSM, based on sex (female or male), HBeAg (positive or negative), nadir CD4+ T cell count (< or ≥200 cells/μL), plasma HIV RNA at CD4 nadir (< or ≥200,000 copies/mL), alanine transferase (ALT) (over local laboratory upper normal limit [derangement] or not) and country (Australia or Thailand). In addition, post-hoc subgroups based on CD4 nadir (%) (< or ≥10%) and ART duration (≤ or >10 years) were explored. Baseline LSM and change over time (kPa), both log base e transformed, were compared for subgroups using robust linear regression, unadjusted and adjusted for CD4+ T-cells at study entry (except for nadir CD4+ T cell count and CD4 nadir [%]).

Detectable virus in plasma was defined as HIV>50 copies/mL or HBV>20 IU/mL. Differences in progressor status between participants with detectable HIV or HBV at any study visit were explored visually and using descriptive statistics.

Analysis included complete cases except for the longitudinal analysis which was based on all available data. Assumptions underlying the statistical models were checked. In particular, the linearity assumption underlying the logistic regression models was tested by comparing first-order and second-order fractional polynomial functions with the linear function.^27^ The more complex model was selected only if this improved the fit statistically at the 5% significance level. Normality and homogeneity underlying the linear regression models as well as outlying or influential values were assessed by (visual) inspection of the residuals. Sensitivity analyses were performed if major violations of assumptions were identified. A two-sided 5% level of significance was used, without multiple testing adjustment due to the exploratory nature of the analysis. STATA/SE Version 15.1 for Windows and Origin (Pro), Version 2021, OriginLab Corporation, Northampton, MA was used.

The statistical analysis plan for the study is included as a Supplementary file.

#### Role of the funding source

The funder had no role in the study design, data collection, data analysis, interpretation, writing of this manuscript or in the decision to submit the paper for publication.

## Results

### Clinical characteristics at enrolment

We identified 68 of 72 eligible participants (Supplementary Figure S1, Table 1). One participant was lost to follow-up after the study entry visit, so was excluded from any analysis. Median age of the 67 participants was 49 years and 85% were male. Alcohol was not used at all by 37% of participants and a further 30% drank 1–2 drinks less often than monthly. 10% (n = 7) drank alcohol daily or almost daily and of this group, most (70%) drank 3–4 drinks or more on a typical day (Supplementary Table S1).

**Table 1:** Baseline characteristics in total and by site.

	Total	Australia	Thailand
General			
Numbers, n (%)	67 (100%)	36 (54%)	31 (46%)
Age, years	49.3 (44.0–58.1)	52.9 (49.0–62.7)	44.7 (41.2–49.3)
Female, n, (%)	10 (15%)	1 (3%)	9 (29%)
Excess alcohol^a^ n, (%)^a^	8 (12%)	7 (19%)	1 (3%)
BMI, kg/m^2^, mean (SD)	23.5 (3.7)^a^	24.0 (3.9)^b^	22.9 (3.3)
HBV			
HBeAg positive, n (%)	21 (31%)	13 (36%)	8 (26%)
ALT, U/L	29.0 (22.0–37.0)	28.0 (21.0–38.5)	32.0 (22.0–36.0)
ALT derangement^b^, n (%)	9 (13%)	7 (19%)	2 (6%)
Level (xULN)	1.4 (1.1–1.7)	0.7 (0.6–1.0)	0.6 (0.4–0.7)
HIV			
Duration ART ≤10 years, n (%)	36 (54%)	14 (39%)	22 (71%)
Time on ART, years	10 (8–16)	12 (7–17)	9 (8–13)
Nadir CD4+ T-cell count, cells/*μ*L	128.0 (28.0–230.0)	135.0 (21.0–246.5)	88.0 (34.0–226.0)
Nadir CD4+ T-cells, %	10.0 (4.0–15.0)^c^	10.5 (4.0–16.0)^d^	10.0 (2.0–14.0)
Nadir CD4+ T-cell count <200 cells/*μ*L, n (%)	43 (64%)	23 (64%)	20 (65%)
Nadir CD4 <10%, n (%)	28 (44%)^c^	14 (44%)^d^	14 (45%)^i^
HIV RNA at nadir CD4, log 10 copies/mL	4.7 (3.8–5.0)^e^	4.1 (3.1–5.0)^f^	4.8 (4.2–5.1)
HIV RNA ≥200,000 copies/mL at nadir, n (%)	9 (14%)^e^	3 (9%)^f^	6 (19%)
CD4+ T-cell count, cells/*μ*L	534 (389–804)	465 (318–761)	577 (475–811)
CD4+ T-cells, %	28 (22–35)	25.5 (20.5–35)	29 (25–35)
CD8+ T-cell count, cells/*μ*L	760 (536–1035)	741.5 (528.5–1197)	767 (642–1007)
Treatment			
2 HBV-active agents, n (%)	62 (93%)	31 (86%)	31 (100%)
<2 HBV-active agents, n (%)^c^	5 (7%)	5 (14%)	0 (0%)
Liver fibrosis			
Fibrosis—LSM by TE, kPa	5.0 (4.3–5.8)^g^	4.9 (4.4–6.3)^h^	5.1 (4.2–5.7)
Fibrosis—Metavir stage equivalent (n, %)	^g^	^h^	
F1 (<5.9 kPa)	48 (75%)	24 (73%)	24 (77%)
F2 (5.9–7.5 kPa)	6 (9%)	3 (9%)	3 (10%)
F3 (7.6–9.3 kPa)	5 (8%)	3 (9%)	2 (6%)
F4 (≥9.4 kPa)	5 (8%)	3 (9%)	2 (6%)
TE, Fibrosis classified			
Mild (F1, F2)	54 (84%)	27 (82%)	27 (87%)
Severe (F3, F4)	10 (16%)	6 (18%)	4 (13%)
Cytokines			
IP10 (CXCL10), pg/mL	9.2 (4.3–24.2)	7.8 (4.7–27.1)	10.2 (3.7–21.1)
HMGB1, ng/mL	2.7 (1.9–4.2)	2.6 (1.6–5.2)	2.9 (1.9–3.7)

All data are presented as median (IQR) unless indicated. SD, Standard Deviation, IQR, Interquartile range. Total n = 67, Australian n = 36 and Thailand n = 31, unless indicated.

LSM, liver stiffness measurement, TE, transient elastography, HMGB1, high mobility group box 1 protein. ^a^n = 66, ^b^n = 35, ^c^n = 63, ^d^n = 32, ^e^n = 65, ^f^n = 34, ^g^n = 64, ^h^n = 33, ^i^n = 31 (Liver fibrosis data was missing for 3 participants from Australian sites due to no baseline scan available).

a Excess alcohol’ indicates at least one of “how often have you had alcoholic drinks” = daily/almost daily OR “number of standard drinks on a typical day when you are drinking” = 10 or more OR “over past 6 months, how often have you had ≥ six drinks on one occasion” = daily/almost daily. Further details are in Supplementary Table S2.

b ALT derangement defined as outside of local laboratory reference ranges which differed by site: in Thailand ALT <55U/L, AST <35U/L; Melbourne <40U/L, AST <35U/L; Sydney ALT <30U/L, AST <30U/L.

c <2 HBV-active agents (n = 4 lamivudine (3TC) only and n = 1 nil HBV active medication).

**Fig. 1:**
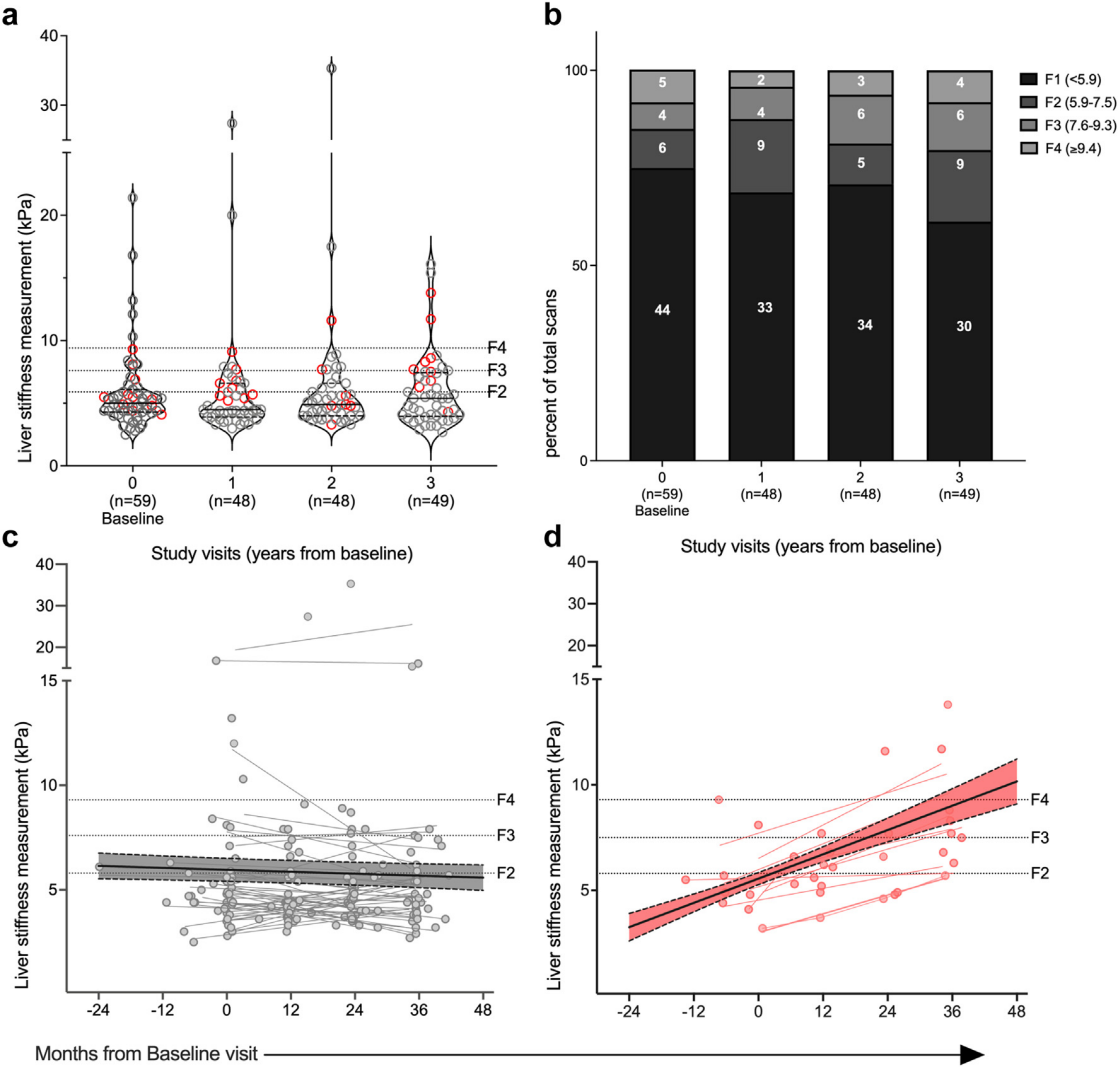
**Liver fibrosis of study participants over time.** Liver stiffness measurements (LSM) were assessed using transient elastography (TE) in each participant at baseline and over 48 weeks follow up at three visits. In panel (a) LSM measured in kPa is shown for each participant with red symbols representing results for progressors (n = 11) (defined as increase in Metavir stage equivalent). The cut off for fibrosis grade F2–4 is shown as a dotted line. Violin plots show the median (horizontal black line) and interquartile range (horizontal dashed black line). Panel (b) shows the proportion of participants at each study visit with LSM scores defined by grade; In panel (c) linear regression of LSM measurements over time (95% confidence intervals) for non-progressors (n = 48) and (d) progressors (n = 11) (both defined by change in Metavir score). Baseline LSM was included with a window from −2 years to +6 months after enrolment. Visits 1, 2 and 3 were defined with a window ± 6 months around 12, 24 and 36 months respectively. Fibrosis definitions were F1 < 5.9 kPa (kilopascals), F2 5.9–7.5 kPa, F3 7.6–9.3 kPa, F4 ≥ 9.4 kPa as previously defined for people living with HIV-HBV co-infection.^20^^,^^21^

A nadir CD4+ T-cell count less than 200 cells/μL was seen in 64% of participants, 31% were HBeAg positive and 13% had ALT above the upper normal limit. Median LSM was 5.0 kPa (IQR: 4.3–5.8), 75% of participants had fibrosis < F2 and 16% had severe liver fibrosis (≥F3) (Table 1, Fig. 1). Median ART duration was 10 years (IQR: 8–16). At baseline, 93% (62/67) were taking tenofovir disoproxil (TDF) and emtricitabine or lamivudine (FTC/3TC), whilst an additional 4 were taking FTC/3TC but not tenofovir (summarised in Supplementary Table S2). One participant was not on an HBV active agent (on raltegravir, etravirine and darunavir) but was still HBsAg positive with suppressed Hepatitis B DNA, thus meeting eligibility criteria. During the study, ART was changed in 14 participants (21%), two of whom changed twice (full details in Supplementary Table S2). Only one participant received tenofovir alafenamide, with the remainder taking tenofovir disoproxil.

Compared to Australian participants, Thai participants (n = 31) were younger, more likely to be female, more commonly on ART for ≤10 years, had lower median nadir CD4+ T cells, higher CD4+ T-cells at enrolment and higher pre-ART HIV RNA (Table 1). Thai participants were more likely to be on two HBV-active agents. Baseline LSM were similar and no evidence of differences by site were seen in plasma cytokines (Supplementary Table S3).

The only difference in liver fibrosis by subgroup was baseline LSM which was higher amongst men than women (median (IQR) = 5.1 (4.4–6.1) and 4.4 (3.2–4.4) respectively) (Supplementary Table S4).

### Changes in liver stiffness over time

**Supplementary Figure S2:**
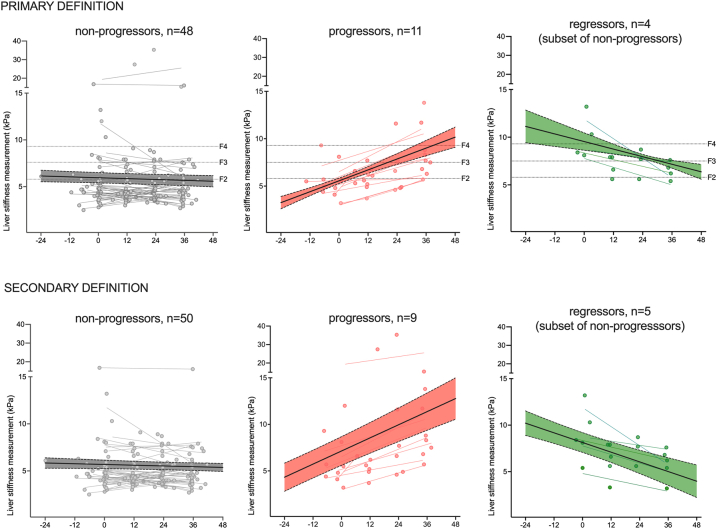
**Liver fibrosis of study participants over time—**Linear regression of LSM measurements over time with 95% confidence intervals for non-progressors (left), progressors (middle) and regressors (subset of non-progressors), using the pre-determined primary definition (increase in Metavir stage equivalent from baseline to final scan) (upper panel) and secondary definition (fibrosis progression of 20% increase and greater than or equal to LSM 2kPa with at least one value over 5·9 kPa) (lower panels), and regressors defined as an analogous relative decrease in LSM [22]. In the upper panels, horizontal dotted lines indicate elastography breakpoints for Metavir stages. Baseline LSM was included with a window from −2 years to + 6 months after enrolment. Visits 1, 2 and 3 were defined with a window +/− 6 months around 12, 24 and 36 months respectively.

Amongst the participants, 59/67 had at least one LSM allowing assessment of fibrosis progression (Supplementary Figure S1, Fig. 1a). Using the primary definition, 11/59 (19%) participants had increased Metavir stage during 3-years or 6/100py after 175py of follow-up. A small number of participants (5/59) had stage 4 fibrosis (LSM ≥9.4 kPa) at study entry, thus could not be assessed for progression. When these participants were removed, 11/54 (20%) were progressors during 3-years or 7/100py after 158py follow-up (Fig. 1b, Supplementary Figure S2). Using our pre-defined secondary definition of liver disease progression (increase LSM by ≥2 kPa), 9/59 (15%) were progressors during 3-years or 5/100py after 175py follow-up (Supplementary Figure S2). Eight participants were progressors by both definitions.

Median (IQR) change LSM over time overall was 0.0 (−1.2 to 1.6) kPa after 175py of follow-up over a median (IQR) 35 (34–41) months. This didn't change when adjusted for baseline CD4+ T-cells (Supplementary Table S5). By subgroup comparisons, the only statistical difference in change in LSM over time was in those with CD4+ T-cell nadir <10% who had a bigger LSM increase compared to those with CD4 T-cell nadir >10% (Supplementary Table S6).

A 14% (95% CI: 7%–21%) average kPa increase every 12-months was seen amongst progressors (Supplementary Table S7). Fibrosis regression occurred in 4 participants or 25% (4/16) with stage >F1 at baseline during 3-years or 8/100py follow-up (Supplementary Figure S2).

### Characteristics and predictors of liver disease progression on ART

**Fig. 2:**
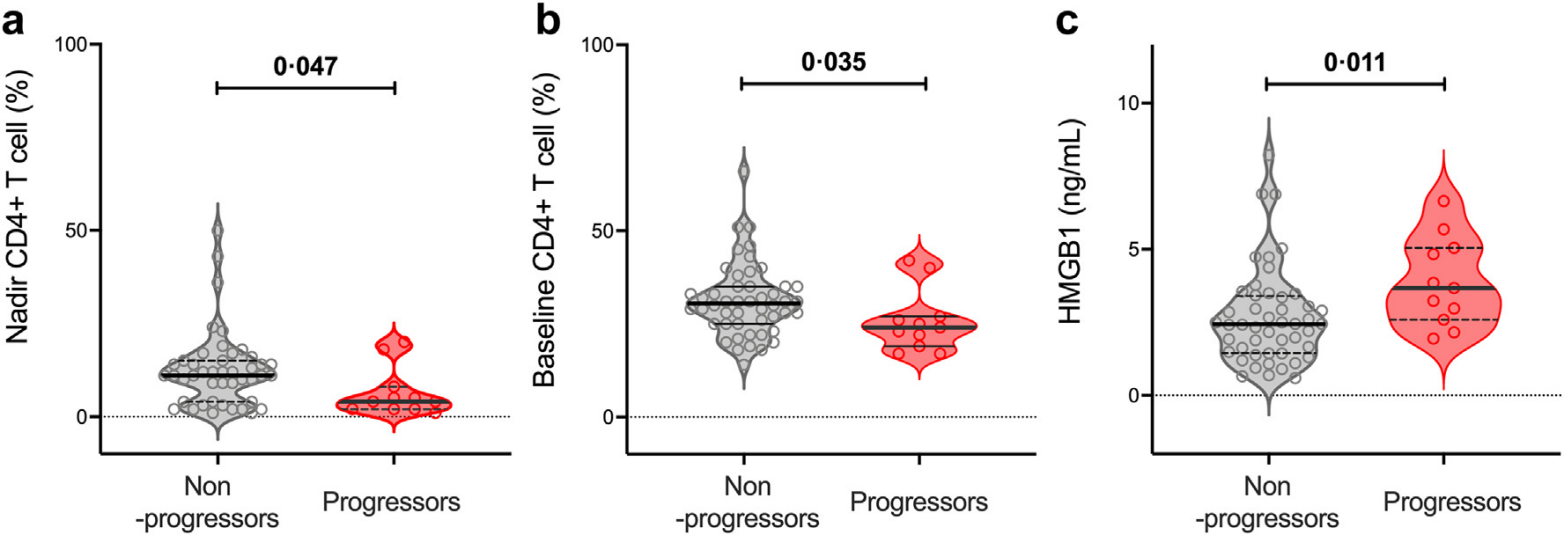
**Clinical parameters that differed at baseline between progressors and non-progressors.** Panels show results for non-progressors (black) and progressors (red) for (a) CD4 T-cell nadir percentage (n = 44 non-progressors, n = 11 progressors), (b) current CD4 T-cell percentage (n = 48 non-progressors and 11 progressors), and (c) high mobility group box 1 (HMGB1) (n = 48 non-progressors and 11 progressors); Each participant is represented by a symbol within a violin plot showing the median (horizontal black line) and interquartile range (horizontal dashed black line). Statistical comparisons were made using Wilcoxon rank sum test with relevant p-values shown.

Progressors had lower nadir CD4+ T cells and percentage, lower current CD4+ T cell percentage and higher HMGB1 (Table 2, Fig. 2, Supplementary Table S8). In regression analysis, progressors compared to non-progressors had higher levels of HGMB1 (by grade: adjusted relative risk (aRR) 1.47, 95% confidence interval (CI) [1.00, 2.17]; by kPa: aRR 1.50, 95% CI [0.98, 2.28]) and lower nadir CD4+ T-cell percentage (by grade: RR 0.93, 95% CI [0.88, 0.98]; by kPa: RR 0.91, 95% CI [0.85, 0.98]). There was no difference in other markers of inflammation by progressor status (Supplementary Table S9).

**Table 2:** Baseline characteristics by progressor status (grade).

	Progressor status (Grade change)		Unadjusted p-value	Adjusted relative risk^c^ estimate (95% CI)
	No	Yes		
**General**				
Numbers, n (%)	48 (81%)	11 (19%)	–	–
Site, n (%)			0.41	
Australia	24 (50%)	4 (36%)		Reference level
Thailand	24 (50%)	7 (64%)		1.73 (0.55, 5.38)
Age, years	47.7 (44.0–53.8)	52.3 (43.3–58.1)	0.70	1.00 (0.94, 1.06)
Sex, n (%)			1.00	
Male	40 (83%)	10 (91%)		Reference level
Female	8 (17%)	1 (9%)		0.57 (0.08, 3.86)
Alcohol, n (%)^a^			1.00	
No excess	43 (90%)	10 (91%)		Reference level
Excess	5 (10%)	1 (9%)		0.76 (0.11, 5.21)
BMI, kg/m^2^ (mean, SD)	23.5 (3.3)^a^	21.7 (3.7)	0.16	0.98 (0.93, 1.04)
**HBV**				
HBeAg, n (%)			1.00	
Negative	32 (67%)	8 (73%)		Reference level
Positive	16 (33%)	3 (27%)		0.76 (0.23, 2.56)
ALT, U/L	28 (20–36)	33 (27–42)	0.20	1.01 (0.98, 1.05)
ALT derangement, n (%)			1.00	
No	41 (85%)	10 (91%)		Reference level
Yes	7 (15%)	1 (9%)		0.61 (0.09, 4.14)
**HIV**				
Duration ART, n (%)			0.49	
>10 years	23 (48%)	4 (36%)		Reference level
≤10 years	25 (52%)	7 (64%)		1.32 (0.42, 4.17)
Time on ART, years	10 (8–15)	9 (8–17)	0.63	0.99 (0.89, 1.10)
Nadir CD4+ T-cell count, cells/*μ*L	135.0 (36.5–228.0)	59.0 (20.0–247.0)	0.37	1.00 (0.99, 1.00)
Nadir CD4+ T-cells, %	11.0 (4.0–15.0)^b^	4.0 (2.0–8.0)	0.047	0.93 (0.88, 0.98)
Nadir CD4+ T-cell count, n (%)			0.73	
≥200 cells/*μ*L	17 (35%)	3 (27%)		Reference level
<200 cells/**μ**L	31 (65%)	8 (73%)		1.37 (0.41, 4.60)
Nadir CD4+ T-cells, n (%)			0.006	
≥10%	29 (66%)^b^	2 (18%)		Reference level
<10%	15 (34%)^b^	9 (82%)		5.81 (1.38, 24.45)
HIV RNA (log 10 cps/mL) at nadir CD4	4.7 (3.9–5.0)^c^	4.7 (3.8–5.3)	0.83	0.99 (0.61, 1.61)
HIV RNA at nadir, n (%)			0.64	
<200,000 cps/mL	40 (87%)^c^	9 (82%)		Reference level
≥200,000 cps/mL	6 (13%)^c^	2 (18%)		1.36 (0.36, 5.14)
CD4+ T-cell count, cells/*μ*L	568 (393–813)	517 (453–653)	0.57	1.00 (1.00, 1.00)
CD4+ T-cell count, %	30.5 (25–35)	24 (19–27)	0.035	0.97 (0.96, 0.99)
CD8+ T cell count, cells/*μ*L	737 (531–981.5)	932 (712–1469)	0.094	1.00 (1.00, 1.00)
**Treatment**				
2 HBV-active agents, n (%)	46 (96%)	9 (82%)	0.15	Reference level
<2 HBV-active agents, n (%)^b^	2 (4%)	2 (18%)		2.83 (0.82, 9.74)
**Liver fibrosis**				
**Fibrosis—LSM by TE, kPa**	4.8 (4.2–6.0)	5.5 (4.8–6.9)	0.25	0.98 (0.84, 1.15)
**Fibrosis—Metavir stage equivalent (n, %)**			0.45	
F1 (<5.9 kPa)	35 (73%)	8 (73%)		Reference level
F2 (5.9–7.5 kPa)	5 (10%)	1 (9%)		0.98 (0.15, 6.33)
F3 (7.6–9.3 kPa)	3 (6%)	2 (18%)		2.22 (0.66, 7.43)
F4 (≥9.4 kPa)	5 (10%)	0 (0%)		–
**TE, Fibrosis—mild or severe**			1.00	
Mild (F1, F2)	40 (83%)	9 (82%)		Reference level
Severe (F3, F4)	8 (17%)	2 (18%)		1.00 (0.25, 4.03)
**Cytokines**				
IP10 (CXCL10), pg/mL	7.0 (4.2–21.3)	8.3 (3.4–12.5)	0.51	0.98 (0.95, 1.02)
HMGB1, ng/mL	2.4 (1.5–3.4)	3.7 (2.6–5.0)	0.011	1.47 (1.00, 2.17)

All data are presented as median (IQR) unless indicated. SD, Standard Deviation, IQR, Interquartile range (25th–75th percentile), CI, Confidence Interval, LSM, liver stiffness measurement, TE, transient elastography, HMGB1, high mobility group box 1 protein.

Eight participants were unable to be classified as progressor or non-progressor due to having only one LSM. Missing data occurred for ^a^n = 47, ^b^n = 44, ^c^n = 46. One of these was on <2 HBV-active agents.

a Excess alcohol’ indicates at least one of “how often have you had alcoholic drinks” = daily/almost daily OR “number of standard drinks on a typical day when you are drinking” = 10 or more OR “over past 6 months, how often have you had ≥ six drinks on one occasion” = daily/almost daily.

b <2 HBV-active agents (n = 3 lamivudine (3TC) only and n = 1 nil HBV active medication).

c After adjusting the comparison between progressors and non-progressors for baseline CD4 count for all characteristics (except for Nadir CD4+ T-cell count, cells/*μ*L, Nadir CD4+ T-cell, %, Nadir CD4 <200+ T-cell count, cells/*μ*L (n,%), CD4+ T-cell count, cells/*μ*L, CD4+ T-cell, %) using logistic regression with a linear relationship between each continuous characteristic and the logit.

Nadir CD4+ T cell percentage <10% was more common in progressors compared to non-progressors, (when liver fibrosis was defined either by 9/11 (82%) versus 15/44 (34%) stage [Table 2], or 8/9 (89%) versus 16/46 (35%) by kPa [Supplementary Table S8]). There was no evidence of differences in progressor status by other baseline characteristics including those associated with excess alcohol intake (Table 2).

### Change in clinical and inflammatory parameters over time in progressors and non-progressors

#### BMI

Over the course of the study, the median (IQR) change in weight and BMI was 0.7 kg (−1.3 to 3.23) and 0.24 kg/m^2^ (−0.48 to 1.16) respectively and there were no observed differences between progressors and non-progressors (weight: −0.3 kg (−3.3 to 2.6) versus 0.8 kg (−1.35 to 4.1), BMI: −0.11 kg/m^2^ (−1.13 to 0.84) versus 0.28 kg/m^2^ (−0.52 to 1.54)).

#### Virological control (plasma)

**Supplementary Figure S3:**
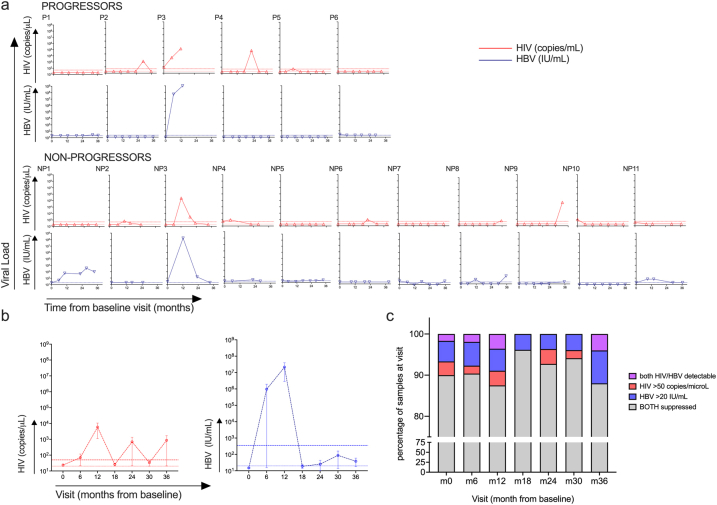
**HIV and HBV viral suppression over the course of the study was present in most individuals, with (a)** Plasma viral load for HIV RNA (red) and HBV DNA (blue) shown for participants with detectable virus on at least one occasion of either HIV (>50 copies/mL)) or HBV (>20 IU/mL) or both over the study duration in progressors (upper rows, P1–7) and non-progressor (lower rows, NP1–9). Each column represents one participant. (**b)** HIV (upper panel) and HBV (lower panel) viral load (mean +95% confidence interval) amongst all participants for each study visit; (**c)** The percentage of subjects at each visit with both HIV>50 copies /mL and HBV >20 IU/mL—grey; with HIV>50 copies/mL and HBV <20 IU/mL (red); with HBV >20 IU/mL and HIV <50 copies/ mL (blue); with HBV >20 IU/mL and HIV >50 copies/mL (purple).

A post-hoc analysis was performed to understand the relationship between virological control and fibrosis progression. Most participants had stable suppressed HIV RNA and HBV DNA in plasma. However, 18 participants had detectable HIV RNA (n = 12) and/or HBV DNA (n = 12) at least once (Supplementary Figure S3). Five had detectable HIV RNA (>50 copies/mL) and HBV DNA (>20 copies/mL). Adherence data were collected (reported missed doses) but were incomplete. In one case a significant number of missed doses corresponded to increasing detectable virus and this participant withdrew from the study. Most detectable virus was low level and not sustained. Of the samples with detectable HIV RNA, 75% (9/12) were only detectable on one sample for a single participant. The median level of detectable HIV was 104 copies/mL (IQR 69–2735 copies/mL). Repeated detectable virus was more common for HBV with 50% (6/12) of participants having detectable HBV DNA on two measurements and 25% (3/12) more frequently. The median level of detectable HBV was 48 (IQR 28–483 copies/mL).

By primary definition, the proportion of progressors amongst participants with detectable HIV RNA on at least one occasion was 4/11 (36%) compared to 7/48 (15%) with suppressed HIV RNA at every visit. The proportion of progressors, amongst participants with detectable HBV DNA on at least one occasion, was 3/12 (25%) compared to 8/47 (17%) with suppressed HBV DNA at every visit. Of participants with detectable HIV RNA and/or HBV DNA at least once, 6/18 (33%) were progressors (by stage) compared to 5/41 (12%) with both viruses fully suppressed. Similar results were found using the secondary definition of progressors (by kPa).

#### HBeAg and HBsAg seroconversion

Changes in HBV serology were similar in progressors compared with non-progressors. HBsAg was lost in one progressor and one non-progressor, without detection of anti-HBsAb. HBeAg seroconversion with gain of anti-HBeAb was seen in one progressor. Loss of HBeAg occurred in one non-progressor (without anti-HBeAb). In one progressor and one non-progressor, HBeAg (without anti-HBeAb) went from negative to positive. Transient changes in HBeAg detection were seen in one progressor and three non-progressors.

#### Immunological, inflammatory, and apoptotic parameters

Using a longitudinal analysis model for the whole cohort, there were no changes in liver enzymes and no change in CD4+ T cells over time. A decrease in CD8+ T cell count occurred (estimate 3% (95% CI: 0–5%) but this is unlikely to be clinically significant (Supplementary Table S5). There were decreases in CXCL10, CXCL11, CCL2, CCL3, CCL4, CCL5, TNF-α, IL10 and IL18 (Supplementary Table S5) but no change in sCD14. HMGB1 increased (change estimate 10% (95% CI: 2–18%) annually (Supplementary Table S5). There was no difference in 12-monthly rate of change over time by progressor status for immunological, inflammatory, or apoptotic markers (Supplementary Table S7).

## Discussion

We found liver fibrosis progression in PWH and HBV co-infection in 20% in those without F4 fibrosis at baseline, despite suppressive HBV-active ART. This contrasts with HBV mono-infection where fibrosis progression on antiviral treatment is uncommon.^7^^,^^8^ Fibrosis progression was associated with elevated HMGB1 and lower nadir CD4+ T-cell count. Regression occurred in 4 participants or 25% (4/16) of those with stage >F1 at baseline during 3-years or 8/100py over 49py follow-up (Supplementary Figure S2).

Only one previous study has prospectively examined liver fibrosis progression in participants already established on stable on suppressive ART, mainly containing tenofovir with similar follow-up duration.^17^ In this study from North America, liver disease progression (increase of ≥2 Ishak fibrosis score points, assessed by biopsy (n = 139, 62 with paired biopsies)) occurred in 12%, whilst 7% had improvement. In our study, we observed liver disease progression in 19% of participants (7/100py follow-up). The use of liver biopsy as well as a different scale and higher threshold for definition of progression in this prior study^17^ may explain the slightly lower rate of liver disease progression observed. Age and sex distribution were similar; however, fewer participants had detectable HBeAg. The study populations may also have differed in sequence of HIV and HBV infection and age of HBeAg seroconversion, given in our study most participants were recruited in Asia where vertical transmission of HBV is generally more common than in North America.^28^ Other potential factors including alcohol use patterns and BMI were not found to be associated with fibrosis in either study but are important to consider as possible confounders.

A very interesting finding in our study was that progressors had significantly higher levels of the damage associated molecular protein (DAMP), HMGB1 at enrolment. HMGB1 is ubiquitously expressed at high level in parenchymal and non-parenchymal liver cells. It is passively released during cell death and may also be actively secreted as a soluble protein during cellular stress or tissue injury.^29^ We only measured circulating HMGB1 and therefore are unable to identify the source of HMGB1. HMGB1 has been identified previously as an important marker and mediator of liver disease pathogenesis in HBV mono-infection,^30^ with a range of roles demonstrated in vitro and murine studies including stellate cell activation and fibrogenesis.^31^^,^^32^ In a recent study we found increased levels of HMGB1 in PWH with HBV coinfection compared with those with HBV alone, with higher levels seen in those with more severe fibrosis. In an in vitro model we also demonstrated that exposure to sera from PWH and HBV coinfection enhanced activation of primary hepatic stellate cells, the key mediator of fibrosis in the liver.^33^ A previous study by our group found increased levels of apoptotic hepatocytes in liver biopsies from PWH with HBV co-infection compared with those who had HBV mono-infection.^34^ Elevated circulating HMGB1 in PWH with HBV co-infection who are progressors could be related to higher levels of hepatocyte death, however, we are unable to confirm this in an observational study. A larger study is needed to fully determine the clinical utility of HGMB1 in predicting liver disease progression. This larger study could also address the important issues of sensitivity, specificity, positive predictive value, and negative predictive value.

Immune suppression was also found to be associated with liver disease progression. CD4 nadir percentage but not absolute nadir or CD4 nadir <200 were significantly associated with fibrosis progression. CD4% will vary with changes in total CD8 T-cells, so it is possible that the CD4 percentage reflects both immunosuppression (loss of CD4+ T-cells) as well as generalised inflammation (reflected as a high CD8+ T-cell count). An association between lower baseline CD4+ T-cells and liver fibrosis stage has been previously described in PWH and HBV.^5^^,^^9^^,^^13^^,^^15^^,^^19^^,^^35^ Lower CD4+ T-cell nadir is associated with poorer recovery of CD4+ T-cells, although in our study, current CD4+ T-cells were not different between progressors and non-progressors. Lower CD4+ T-cell nadir has also been associated with increased CD4+ T-cell turnover^36^ and therefore increased cellular proliferation and death, which could also have been a source of circulating HMGB1 amongst progressors in our study. Finally, advanced immunosuppression may accelerate liver fibrosis through increased translocation of microbial products due to lymphocyte depletion from the gastrointestinal mucosa, or persistent expression of pro-inflammatory cytokines.^25^^,^^37^^,^^38^ However, in our study, circulating markers of inflammation or microbial translocation were not associated with liver disease progression.

Strengths of our study were that participants were on stable suppressive ART at study enrolment, and there were high rates of study retention and prolonged follow up. Limitations include a small sample size limiting the number of potential confounding factors that could be incorporated into modelling such as alcohol intake or hepatitis D infection (which was not available) and precision of the estimated associations. It was not possible to perform intrahepatic sampling or biopsy in this study. LSM is a good alternative to liver biopsy and has been validated in PWH and HBV coinfection.^20^^,^^21^ We recognise that a categorical classification of fibrosis in HIV-HBV co-infection is imperfect with these cut-offs derived with lower specificity to enhance detection of cirrhosis.^20^^,^^21^ However, to overcome this limitation, in our study we used two approaches to measure changes in liver stiffness with both categorical and continuous variables and overall arrived at the same conclusions.

An important strength of our study is its prospective nature and the inclusion criteria of viral load suppression in individuals who were mainly on tenofovir containing ART. In our study, HIV and HBV were suppressed in most participants throughout the study. While we observed liver fibrosis regression in 25% of participants over 3 years, other prospective HIV-HBV studies observed higher rates of fibrosis regression of over 40% using LSM.^9^^,^^12^^,^^14^^,^^15^ These differences in findings may be explained by initiation of ART itself resulting in reduced viral replication and therefore decreased liver inflammatory activity and hence LSM.^9^^,^^12^^,^^14^^,^^15^ Furthermore, suppression of plasma HBV DNA is associated with liver fibrosis regression and is more common after switching to tenofovir containing therapy.^3^^,^^9^^,^^12^ Decreased LSM was associated with high HBV DNA pre-tenofovir.^9^^,^^12^ In HBV mono-infection, a high plasma HBV DNA VL prior to therapy, was the strongest predictor of progression to cirrhosis over 11 years’ follow up.^39^

The possibility of an increase in HIV and HBV viral load leading to higher inflammation and therefore an increase in LSM cannot be excluded, although a direct association was not seen in our study. In a similar prospective cohort study performed in France, liver fibrosis was examined from time of switch to tenofovir based therapy. After a median 32.5 months on tenofovir, 10.5% had a higher Metavir stage on paired liver biopsy (n = 38).^18^ An updated analysis of a larger group (n = 169) including the same cohort after a median 7.6 years, showed fibrosis progression in 22.5%, assessed by ‘Fibrotest’,^19^ consistent with our findings.

The lack of a comparator group with HBV alone may be considered a limitation, however given different thresholds for treatment initiation, comparisons between these two groups are difficult to make. Finally, there were differences at baseline between Australian and Thai participants, with Thai participants being younger and more recently diagnosed with HIV infection. The pattern and natural history of HBV infection in Thailand (endemic HBV and usually acquired in childhood), is different from Australia (where HBV is not endemic and usually acquired as an adult). HBV genotype also differs between these two geographical regions,^40^ but could not be measured in this study. There was, importantly, no difference in fibrosis levels and no difference in HMGB1 nor nadir CD4+ T-cells between Australian and Thai participants.

In summary, in this prospective longitudinal cohort study, liver fibrosis progression occurred in almost one fifth of PWH and HBV co-infection on stable ART, despite suppressive ART. Liver disease progression was associated with lower nadir CD4+ T-cell count and higher levels of HMGB1 at baseline. The source of HMGB1 is unclear but could arise from increased hepatocyte cell death leading to fibrogenesis or enhanced CD4+ T-cell turnover in people who initiate ART with a low CD4+ T-cell count. Our findings of liver fibrosis progression being associated with lower nadir CD4+ T-cell count supports the importance of early and sustained suppressive HBV-active ART in reducing morbidity and mortality in PWH and HBV co-infection.

## Contributors

KPS, AA, MC, JA, SRL conceptualized the study. Data curation was by KS, AA, JZ, WZ, SB, AR, GVM, CKF, JS, MC and JA. Formal analysis was by SB and KPS. Funding acquisition was by MC and SRL. KS, AA, JZ, WZ, SB, GVM, CKF, JS, ST, AR, MC, JA and SRL all contributed to investigation and methodology. Project administration was by KPS, AA, ST, AR, GVM, CKF, JS, MC, JA and SRL. AA, GVM, CKF, JS, MC, JA and SRL supervised the project. KPS, SB, JA and SRL wrote the original draft. KS, AA, JZ, WZ, SB, GVM, CKF, JS, ST, AR, MC, JA and SRL all contributed to review and editing. KS, AA, JZ, WZ, SB, GVM, CKF, JS, ST, AR, MC, JA and SRL all read and approved the final version. KPS, SB and JA all directly accessed and verified the underlying data reported in this manuscript.

## Data sharing statement

The data that support the findings of this study are available on request from the corresponding author.

## Declaration of interests

SRL has received funding for investigator-initiated studies from Gilead Sciences, Merck and Viiv. She has been a member of scientific advisory boards for Gilead Sciences, Merck, Viiv, Abbvie, BMS, Immunocore, Abivax, Vaxxinity, Aelix and Efsam. Honoraria have been paid to her institution and to her personally. None of these activities are on topics related to this manuscript. GM has received grants for research from Gilead Sciences, Abbvie, Janssen and ViiV. She has received consulting fees from Viiv and from Gilead Sciences and has been a member of a scientific advisory board for Viiv. JS has received grants for research from Gilead Sciences, Merck, Moderna, Takeda and GSK. He has been a member of scientific advisory boards for Gilead Sciences, Merck, Astra Zeneca, GSK and Takeda.

## Appendix A Supplementary data

Supplementary Tables S1–S9

Stastical analysis plan
