# Supplementary material for: Predictors of liver disease progression in people living with HIV-HBV co-infection on antiretroviral therapy
Source: eBioMedicine. 2024 Mar 21;102:105054. doi: 10.1016/j.ebiom.2024.105054 (PMC10966452; doi:10.1016/j.ebiom.2024.105054)
Supplement: Supplementary Tables S1–S9 [file mmc1.docx]

**SUPPLEMENTARY TABLES 1-9**

### **Supplementary Table 1 Alcohol intake**

| **Frequency of alcohol intake** | **Number** | **Percentage** | **Number of standard drinks on a typical day** |
| --- | --- | --- | --- |
| **Never** | 25 | 37% | NA |
| **Less than monthly** | 18 | 27% | 13 (72%): 1-2  5 (28%): 3-4 |
| **Monthly** | 6 | 9% | 1 (17%): 1-2  1 (17%): 3-4  2 (33%): 5-6  1 (17%): 7-9  1 (17%): >10 |
| **Weekly** | 11 | 16% | 4 (36%): 1-2  5 (45%): 3-4  1 (9%): 5-6  1 (9%): 7-9 |
| **Daily or almost daily** | 7 | 10% | 2 (29%): 1-2  2 (29%): 3-4  2 (29%): 5-6  1 (14%): 7-9 |

Note: ‘Alcohol excess’ was defined as at least one of “how often have you had alcoholic drinks” =daily/almost daily, “number of standard drinks on a typical day when you are drinking” ≥10 OR “over past 6 months, how often have you had ≥six drinks on one occasion” =daily/almost daily.

**Supplementary Table 2 Antiretroviral therapy taken by participants over the course of the study**

| **Antiretroviral therapy at baseline** | | | | | | | |
| --- | --- | --- | --- | --- | --- | --- | --- |
| **Site, n** | **Tenofovir disoproxil** | **FTC/ 3TC** | **Integrase inhibitor** | **NNRTI** | **PI** | **Other** | **Notes** |
| Thailand,  31 | 31 | 31 | 0 | 27  (EFV (23), NVP (4)) | 5  (ATV/r (3), LPV/r (1), SQV/r (1)) |  | 31/31 on TDF and xTC  2 participants on 4-drug regimens   - TDF/FTC, LPV, ATV/r - TDF/FTC, EFV, SQV/r |
| Australia,  36 | 31 | 34 | 9  raltegravir (8)  DTG (1) | 20  (EFV (5), NVP (9), ETV (5), RPV (1)) | 15  (ATV/r (6), DVR/r ((6), LPV/r (3)) | Abacavir (3),  AZT (2)  Maraviroc (1) | 30/36 on TDF and xTC  1 on TDF without FTC/3TC (raltegravir/ETV)  4 on FTC or 3TC (without TDF)  1 on no HBV-active agent (ETV, raltegravir, DRV/r)  1 on 5-drug regimen   - TDF/FTC, raltegravir, ETV, DRV/r |
| **ART at end of study** | | | | | | | |
| **Site** | **Tenofovir** | **FTC/ 3TC** | **Integrase inhibitor** | **NNRTI** | **PI** | **Other** | **Changes made** |
| Thailand | 31 | 31 | 0 | 28  (EFV (22), NVP (4), RPV (2)) | 4 |  | 1. TDF/FTC/EFV 🡪 TDF/xTC/**RPV** 2. TDF/FTC, ATV/r 🡪 TDF/FTC/**RPV** |
| Australia | 31* | 34 | 11  raltegravir (6)  DTG (3)  Elvitegravir/ cobicistat(2) | 20  (EFV (5), NVP (8), ETV (4), RPV (2)) | 14  (ATV/r (5), DVR/r ((9)) | Abacavir (4)  AZT (1) | 1. TDF/raltegravir/ETV 🡪 TDF/DTG/**RPV** 2. TDF/FTC, LPV/r 🡪 TDF/FTC/**elvitegravir/cobicistat** 3. TDF/FTC, NVP 🡪 TDF/FTC, NVP, **DTG, DRV/r** 4. TDF/FTC/EFV 🡪 TDF/FTC/**RPV** 5. ABC/3TC, LPV/r 🡪 ABC/3TC, **DVR/r** 6. TDF/FTC, LPV/r 🡪 raltegravir**, ETV** 7. 3TC, raltegravir, ETV, DRV/r, maraviroc 🡪 3TC, raltegravir, ETV, DRV/r 8. TDF/FTC, ATV/r 🡪 TDF/FTC, **DRV/r** 9. TDF, AZT/3TC, NVP 🡪 TDF/FTC, **DTG** 10. TDF/FTC, raltegravir, ETV, DRV/r 🡪 TDF/FTC, raltegravir, ETV🡪 **TAF**/FTC, raltegravir 11. TDF/FTC, raltegravir🡪 TDF/FTC, **DTG🡪 ABC/3TC/**DTG 12. TDF/FTC/RPV 🡪 TDF/FTC/**elvitegravir/cobicistat** |

* 1 tenofovir alafenamide (TAF)

Bolded = newly added agent

TDF tenofovir disoproxil fumarate, xTC lamivudine (3TC) or emtricitabine (FTC), ABC abacavir, ATV/r atazanavir/ritonavir, EFV efavirenz, NVP nevirapine, RPV rilpivirine, ATV/r atazanavir/ritonavir, ETV etravirine, DTG dolutegravir, DRV/r darunavir/ritonavir, TAF tenofovir alafenamide

### **Supplementary Table 3 Baseline plasma markers total and by site**

| **Plasma Biomarkers** | **TOTAL (n=67)** | **AUSTRALIA (n=36)** | **THAILAND (n=31)** |
| --- | --- | --- | --- |
| sCD14, pg/mL | 580·4 (459·7-753·4) | 535·9 (433·4-695·4) | 664·5 (522·4-809·3) |
| TNF-⍺, pg/mL | 4·8 (2·8-15·7) | 9·5 (4·8-30·1) | 3·1 (2·1-4·8) |
| IL 10, pg/mL | 1·6 (1·1-2·1) | 1·6 (1·1-2·6) | 1·4 (1·2-2·0) |
| IL 18, pg/mL | 30·2 (16·6-53·5) | 31·3 (19·6-59·1) | 28·2 (12·1-51·0) |
| CXCL-9, pg/mL | 349·9 (248·9-639·1) | 457·2 (318·8-746·8) | 270·8 (232·6-374·7) |
| CXCL10, pg/mL | 9·2 (4·3-24·2) | 7·8 (4·7-27·1) | 10·2 (3·7-21·1) |
| CXCL-11, pg/mL | 5160·3 (3526·9-7316·4) | 5532·2 (3362·5-7335·2) | 4921·5 (3526·9-7316·4) |
| CCL-2, pg/mL | 72·1 (36·9-141·2) | 54·2 (36·6-90·2) | 80·5 (37·6-209·0) |
| CCL-3, pg/mL | 20·1 (12·5-41·1) | 37·1 (17·2-225·4) | 18·1 (11·9-23·2) |
| CCL-4, pg/mL | 68·5 (50·2-187·7) | 112·4 (58·9-867·0) | 55·4 (42·6-72·1) |
| CCL-5, pg/mL | 5887·0 (3729·5-8545·5) | 6418·8 (3591·5-7878·3) | 4870·5 (4059·0-8854·5) |

All data are presented as median (IQR), IQR = Interquartile range (25^th^ to 75^th^ percentile).

**Supplementary Table 4 Differences in liver stiffness measurement (LSM, by TE, kPa) at baseline by subgroup**

|  | No | Yes | p-value  unadjusted^a^ | p-value  adjusted^b^ |
| --- | --- | --- | --- | --- |
| Site - Thailand | 4·9  (4·4-6·3),  n=33 | 5·1  (4·2-5·7),  n=31 | 0·75 | 0·60 |
| Gender – Female | 5·1  (4·4-6·1),  n=55 | 4·4  (3·2-4·4),  n=9 | 0·041 | 0·041 |
| HBeAg positive | 5·3  (4·3-5·9),  n=43 | 4·8  (4·1-5·6),  n=21 | 0·51 | 0·58 |
| CD4+ T-cell count nadir  <200 cells/𝜇L | 5·2  (4·2- 6·9),  n=22 | 4·85  (4·4-5·7),  n=42 | 0·41 | NA |
| CD4+ T-cell nadir (%)  <10% | 5·1  (4·4-5·8),  n=33 | 4·9  (4·1-5·9),  n=27 | 0·67 | NA |
| HIV RNA at CD4 nadir  ≥200,000 cps/mL | 4·9  (4·4-5·8),  n=53 | 5·3  (4·1-5·4),  n=9 | 0·57 | 0·59 |
| Time on ART  >10 years | 5·3  (4·2-8·0),  n=35 | 4·9  (4·3-5·4),  n=29 | 0·37 | 0·21 |
| ALT derangement | 4·9  (4·3-5·8),  n=56 | 5·6  (4·7-6·8),  n=8 | 0·51 | 0·49 |

### All data are presented as median (IQR) and sample size (n). IQR = Interquartile range (25^th^ to 75^th^ percentile), NA = Not Applicable, LSM = liver stiffness measurement, TE = transient elastography. ^a^ Robust linear regression (baseline kPa, log-transformed) with subgroup as covariate. ^b^Adjusted for baseline CD4+ T-cell count.

**Supplementary Table 5 Rate of change over time (linear)**

| **Outcome** | **Rate of change over time (linear)** | |
| --- | --- | --- |
|  | **Estimate (95% CI)** | **p-value** |
| sCD14, pg/ml | 1·02 (0·99,1·05) | 0·30 |
| HMGB1, ng/mL | 1·10 (1·02,1·18) | 0·0085 |
| TNF-⍺, pg/ml | 0·81 (0·74,0·88) | <0·0001 |
| IL-10, pg/ml | 0·93 (0·88,0·98) | 0·0039 |
| IL-18, pg/ml | 0·85 (0·81,0·90) | <0·0001 |
| CXCL-9, pg/ml | 1·00 (0·94,1·06) | 0·88 |
| CXCL-10, pg/ml | 0·83 (0·75,0·92) | 0·0003 |
| CXCL-11, pg/ml | 0·95 (0·91,0·99) | 0·0097 |
| CCL-2, pg/ml | 0·87 (0·83,0·92) | <0·0001 |
| CCL-3, pg/ml | 0·82 (0·75,0·90) | <0·0001 |
| CCL-4, pg/ml | 0·86 (0·79,0·94) | 0·0010 |
| CCL-5, pg/ml | 0·89 (0·85,0·93) | <0·0001 |
| Albumin, g/L* | -0·13 (-0·62,0·35) | 0·59 |
| ALT, U/L | 1·00 (0·96,1·04) | 0·95 |
| AST, U/L | 1·00 (0·97,1·03) | 0·88 |
| CD4+ T-cell count, cells/μL | 1·00 (0·98,1·03) | 0·89 |
| CD4+ T-cells, % | 1·01 (0·99,1·04) | 0·40 |
| CD8+ T-cell count, cells/μL | 0·97 (0·95,1·00) | 0·042 |
| CD8+ T-cells, % | 0·99 (0·97,1·00) | 0·089 |
| CD4:CD8 ratio | 1·03 (1·00,1·06) | 0·057 |
| LSM, kPa | 1·02 (0·99,1·04) | 0·31 |

CI = Confidence Interval, HMGB1 = high mobility group box 1 protein, LSM = liver stiffness measurement. All outcomes were log-transformed, except for albumin. All outcomes were adjusted for baseline CD4+ T-cell count, except for CD4+ T-cell count (cells/𝜇L) and CD4+ T cells (%).

*The analysis of albumin indicated three influential observations (values of 12g/L, 13g/L, and 82g/L while remaining values in the range of 30-52g/L), exclusion of these data points resulted in a 12-monhtly rate of change over time of -0·31 (-0·54,-0·07) with p-value 0·010.

**Supplementary Table 6 Change in liver stiffness measurement (LSM, by TE, kPa), over the course of the study (from first to last LSM) by subgroup**

|  |  | Median (IQR), sample size | p-value unadjusted^a^ | p-value adjusted^b^ |
| --- | --- | --- | --- | --- |
| Site | Thailand | 0·60 (-0·80-1·9), n=31 | 0·60 | 0·56 |
|  | Australia | -0·25 (-1·3-0·9), n=28 |  |  |
| Sex | Female | 1·1 (0·1-2·5), n=9 | 0·24 | 0·25 |
|  | Male | -0·15 (-1·3-1·6), n=50 |  |  |
| HBeAg status | positive | 0·0 (-1·4-1·1), n=19 | 0·44 | 0·43 |
|  | negative | 0·20 (-0·8-1·7), n=40 |  |  |
| CD4+ T-cell count nadir | <200 cells/𝜇L | 0·5 (-0·8, 1·7) n=39 | 0·19 | NA |
|  | ≥200 cells/𝜇L | -0·35 (-1·6, 1·05) n=20 |  |  |
| CD4+ T-cell nadir | <10% | 1·25 (-0·35-2·5), n=24 | 0·0019 | NA |
|  | ≥10% | -0·4 (-1·4-0·6), n=31 |  |  |
| HIV RNA at CD4 nadir | <200,000 copies/mL | 0·0 (-0·8-1·6), n=49 | 0·20 | 0·22 |
|  | ≥200,000 copies/mL | -1·3 (-2·35-1·45), n=8 |  |  |
| Time on ART | <10 years | 0·3 (-0·8-2·45), n=32 | 0·088 | 0·14 |
|  | ≥10 years | 0·0 (-1·3-0·9), n=27 |  |  |
| ALT derangement | Yes | -1·15 (-2·25-0·85), n=8 | 0·46 | 0·42 |
|  | No | 0·0 (-0·8-1·7), n=51 |  |  |

IQR = Interquartile range (25^th^ to 75^th^ percentile), NA=Not Applicable, LSM=liver stiffness measurement, TE=transient elastography. ^a^Robust linear regression (last scan kPa, log-transformed) accounting for first scan (kPa, log-transformed) and subgroup as covariate. ^b^Adjusted for baseline CD4+ T cell count, except for CD4+ T-cell count nadir and CD4+ T cell nadir.

### **Supplementary Table 7 Comparison of rate of change over time in plasma biomarkers and liver stiffness measurement (LSM) between progressors and non-progressors (Grade)**

| **Plasma Biomarkers** | **Progressor (Grade) [yes] slope** | | **Progressor (Grade) [no] slope** | | **Difference in slope [yes] vs [no]** | |
| --- | --- | --- | --- | --- | --- | --- |
|  | **Estimate (95% CI)** | **p-value** | **Estimate (95% CI)** | **p-value** | **Estimate (95% CI)** | **p-value** |
| sCD14, pg/ml | 0·98 (0·91,1·05) | 0·60 | 1·02 (0·98,1·05) | 0·34 | 0·96 (0·89,1·04) | 0·37 |
| HMGB1, ng/mL | 1·05 (0·89,1·23) | 0·58 | 1·16 (1·07,1·25) | 0·0002 | 0·90 (0·75,1·08) | 0·26 |
| TNF-⍺, pg/ml | 0·86 (0·70,1·07) | 0·17 | 0·81 (0·73,0·90) | 0·0001 | 1·06 (0·84,1·34) | 0·63 |
| IL-10, pg/mL | 0·94 (0·83,1·07) | 0·36 | 0·91 (0·86,0·97) | 0·0043 | 1·03 (0·90,1·19) | 0·66 |
| IL-18, pg/mL | 0·89 (0·77,1·02) | 0·084 | 0·84 (0·79,0·90) | <0·0001 | 1·05 (0·90,1·23) | 0·51 |
| CXCL-9, pg/mL | 1·00 (0·85,1·17) | 0·97 | 1·00 (0·93,1·08) | 0·96 | 1·00 (0·84,1·18) | 0·96 |
| CXCL-10, pg/mL | 0·96 (0·75,1·24) | 0·77 | 0·82 (0·73,0·90) | 0·0013 | 1·17 (0·89,1·54) | 0·26 |
| CXCL-11, pg/mL | 0·97 (0·88,1·07) | 0·53 | 0·94 (0·89,0·98) | 0·0094 | 1·03 (0·93,1·15) | 0·56 |
| CCL-2, pg/mL | 0·88 (0·76,1·00) | 0·057 | 0·88 (0·83,0·94) | <0·0001 | 0·99 (0·85,1·16) | 0·93 |
| CCL-3, pg/mL | 0·83 (0·67,1·03) | 0·095 | 0·85 (0·77,0·94) | 0·0022 | 0·98 (0·77,1·24) | 0·85 |
| CCL-4, pg/mL | 0·85 (0·69,1·05) | 0·13 | 0·90 (0·82,1·00) | 0·040 | 0·95 (0·75,1·19) | 0·64 |
| CCL-5, pg/mL | 0·83 (0·75,0·92) | 0·0004 | 0·90 (0·85,0·94) | <0·0001 | 0·93 (0·83,1·04) | 0·18 |
| Albumin, g/L* | 0·27 (-0·96,1·49) | 0·67 | -0·15 (-0·74,0·43) | 0·61 | 0·42 (-0·94,1·78) | 0·54 |
| ALT, U/L | 1·04 (0·94,1·04) | 0·44 | 1·00 (0·95,1·04) | 0·89 | 1·04 (0·94,1·16) | 0·44 |
| AST, U/L | 1·05 (0·98,1·13) | 0·16 | 0·99 (0·96,1·02) | 0·52 | 1·07 (0·98,1·16) | 0·12 |
| CD4+ T-cell count | 1·00 (0·94,1·06) | 0·98 | 1·00 (0·97,1·03) | 0·95 | 1·00 (0·93,1·07) | 0·96 |
| CD4+ T-cells, % | 1·04 (0·98,1·10) | 0·24 | 1·01 (0·98,1·04) | 0·36 | 1·02 (0·96,1·09) | 0·50 |
| CD8+ T-cell count | 0·93 (0·88,0·99) | 0·022 | 0·97 (0·95,1·00) | 0·047 | 0·96 (0·90,1·03) | 0·22 |
| CD8+ T-cells, % | 0·97 (0·93,1·00) | 0·059 | 0·99 (0·97,1·00) | 0·11 | 0·98 (0·94,1·02) | 0·31 |
| CD4:CD8 ratio | 1·07 (1·00,1·15) | 0·097 | 1·03 (1·00,1·07) | 0·075 | 1·03 (0·95,1·12) | 0·46 |
| LSM, kPa | 1·14 (1·07,1·21) | <0·0001 | 0·99 (0·96,1·02) | 0·47 | 1·15 (1·07,1·23) | 0·0001 |

CI = Confidence Interval, HMGB1 = high mobility group box 1 protein, LSM = liver stiffness measurement· All outcomes were log-transformed, except for albumin. All outcomes were adjusted for baseline CD4 count, except for CD4+ cell count (cells/𝜇L) and CD4+ cells (%).

*The analysis of albumin indicated three influential observations (values of 12g/L, 13g/L, and 82g/L while remaining values in the range of 30-52g/L), exclusion of these data points resulted in a 12-monhtly rate of change over time of -0·02 (-0·56,0·52) with p-value 0·94 for progressors and -0·33 (-0·58,-0·07) with p-value 0·012 for non-progressors and their difference 0·31 (-0·29,0·90) with p-value 0·31.

### **Supplementary Table 8 Baseline characteristics by progressor status (kPa)**

|  | **Progressor Status (kPa)** | | **Unadjusted**  **p-value** | **Adjusted relative risk***  **Estimate (95% CI)** |
| --- | --- | --- | --- | --- |
|  | No | Yes |  |  |
| General |  |  |  |  |
| Numbers, n (%)  Site, n (%)  Australia  Thailand  Age, years  Sex, n (%)  Male  Female  Alcohol, n (%) ^†^  No excess  Excess  BMI, kg/m^2^ (mean, SD) | 50 (85%)  24 (48%)  26 (52%)  48·7 (44·0-53·6)  42 (84%)  8 (16%)  45 (90%)  5 (10%)  23·3 (3·4)^a^ | 9 (15%)  4 (44%)  5 (56%)  50·8 (43·3-58·1)  8 (89%)  1 (11%)  8 (89%)  1 (11%)  22·6 (3·7) | -  1·00  0·92  1·00  1·00  0·62 | -  Reference level  1·33 (0·39, 4·61)  0·99 (0·95, 1·03)  Reference level  0·74 (0·11, 5·06)  Reference level  0·86 (0·12, 6·11)  0·97 (0·88, 1·07) |
| HBV |  |  |  |  |
| HBeAg, n (%)  Negative  Positive  ALT, U/L  ALT derangement, n (%)  No  Yes  Duration ART, n (%)  >10 years  ≤10 years  Time on ART, years  Nadir CD4+ T-cell count, cells/𝜇L  Nadir CD4+ T-cells, %  Nadir CD4+ T-cell count, n (%)  ≥200 cells/𝜇L  <200 cells/𝜇L  Nadir CD4+ T-cells, n (%)  ≥10%  <10%  HIV RNA (log10 cps/mL) at nadir CD4 | 34 (68%)  16 (32%)  30·0 (20·0-36·0)  42 (84%)  8 (16%)  25 (50%)  25 (50%)  11 (9-16)  135·0 (39·0-230·0)  11·0 (4·0-15·0)**^b^**  18 (36%)  32 (64%)  30 (65%)^b^  16 (35%)^b^  4·7 (3·9-5·0)**^c^** | 6 (67%)  3 (33%)  27·0 (27·0-39·0)  9 (100%)  0 (0%)  2 (22%)  7 (78%)  9 (8-9)  28·0 (24·0-88·0)  4·0 (2·0-5·0)  2 (22%)  7 (78%)  1 (11%)  8 (89%)  4·9 (4·2-5·3) | 1·00  0·54  0·34  0·16  0·19  0·19  0·034  0·70  0·007  0·54 | Reference level  0·97 (0·27, 3·49)  1·00 (0·96, 1·04)  Reference level  -  Reference level  2·48 (0·55, 11·13)  0·95 (0·86, 1·04)  1·00 (0·99, 1·00)  0·91 (0·85, 0·98)  Reference level  1·79 (0·41, 7·85)  Reference level  10·33 (1·39, 77·07)  1·03 (0·57, 1·87) |
| HIV RNA at nadir, n (%)  <200,000 cps/mL  ≥200,000 cps/mL  CD4+ T-cell count, cells/𝜇L  CD4+ T-cell count, %  CD8+ T-cell, cells/ 𝜇L | 42 (88%)**^c^**  6 (13%)**^c^**  568 (396-815)  30·5 (25-35)  759·5 (531-996) | 7 (78%)  2 (22%)  517 (453-604)  25 (23-26)  824 (659-1209) | 0·60  0·33  0·058  0·60 | Reference level  1·75 (0·45, 6·81)  1·00 (1·00, 1·00)  0·97 (0·95, 0·99)  1·00 (1·00, 1·00) |
| Treatment |  |  |  |  |
| 2 HBV-active agents, n (%)  <2 HBV-active agents, n (%) ^‡^ | 47 (94%)  3 (6%) | 8 (89%)  1 (11%) | 0·49 | Reference level  1·29 (0·18, 9·25) |
| Liver Fibrosis |  |  |  |  |
| Fibrosis – LSM by TE, kPa | 4·9 (4·2-5·9) | 5·5 (4·8-8·1) | 0·20 | 1·03 (0·87, 1·22) |
| Fibrosis – Metavir stage equivalent, n (%)  - F1 (<5·9kPa)  - F2 (5·9-7·5kPa)  - F3 (7·6-9·3 kPa)  - F4 (≥9·4kPa) | 37 (74%)  6 (12%)  3 (6%)  4 (8%) | 6 (67%)  0 (0%)  2 (22%)  1 (11%) | 0·23 | Reference level  -  2·96 (0·86, 10·14)  1·00 (0·13, 7·57) |
| TE, Fibrosis classified  Mild (F1,F2)  Severe (F3,F4) | 43 (86%)  7 (14%) | 6 (67%)  3 (33%) | 0·17 | Reference level  2·16 (0·63, 7·44) |
| Cytokines |  |  |  |  |
| IP10 (CXCL10), pg/mL  HMGB1, ng/mL | 64 (3·7-21·1)  2·5 (1·5-3·4) | 9·2 (5·2-12·5)  3·7 (2·6-5·0) | 0.83  0·032 | 0·99 (0·96, 1·02)  1·50 (0·98, 2·28) |

All data are presented as median (IQR) unless indicated, SD = Standard Deviation, IQR = Interquartile range (25^th^ to 75^th^ percentile), CI = Confidence Interval, LSM = liver stiffness measurement, TE = transient elastography, HMGB1 = high mobility group box 1 protein.

*After adjusting the comparison between progressors and non-progressors for baseline CD4+ T cell count for all characteristics (except for Nadir CD4 count, cells/𝜇L, Nadir CD4, %, Nadir CD4 count <200 cells/𝜇L, CD4 total, cells/𝜇L, CD4 total %) using logistic regression with a linear relationship between each continuous characteristic and the logit.

Eight participants were unable to be classified as progressor or non-progressor due to having only one LSM. Missing data occurred for ^a^n=49, ^b^n=46**,** ^c^n=48.

†Alcohol excess’ indicates at least one of “how often have you had alcoholic drinks” = daily/almost daily OR “number of standard drinks on a typical day when you are drinking” = 10 or more OR “over past 6 months, how often have you had >=six drinks on one occasion” = daily/almost daily.

‡<2 HBV-active agents (n=3 lamivudine (3TC) only and n=1 nil HBV active medication).

**Supplementary Table 9 Other baseline plasma markers by progressor status (Grade) and (kPa)**

|  |  | **By progressor status (Grade)** | | | **By progressor status (kPa)** | | |
| --- | --- | --- | --- | --- | --- | --- | --- |
| **Plasma Biomarkers** | **TOTAL (n=59)** | **Non-progressor (n=48)** | **Progressor (n=11)** | **p- value** | **Non-progressor (n=50)** | **Progressor (n=9)** | **p- value** |
| sCD14, pg/mL | 624·5 (459·7-763·8) | 615·6 (456·8-746·5) | 727·3 (527·3-849·9) | 0·24 | 591·7 (459·3-753·4) | 727·3 (580·4-809·3) | 0·23 |
| TNF-⍺, pg/mL | 4·8 (2·5-8·6) | 4·8 (2·5-7·6) | 3·8 (2·5-23·2) | 0·70 | 4·6 (2·5-8·3) | 4·8 (3·1-23·2) | 0·53 |
| IL 10, pg/mL | 1·4 (1·1-2·1) | 1·4 (1·1-2·1) | 1·6 (1·3-2·6) | 0·51 | 1·5 (1·1-2·1) | 1·4 (0·9-2·0) | 0·92 |
| IL 18, pg/mL | 28·1 (15·0-53·5) | 29·1 (14·8-53·2) | 27·1 (15·0-71·4) | 0·99 | 29·1 (12·1-53·5) | 27·1 (17·9-42·8) | 0·92 |
| CXCL-9, pg/mL | 339·2 (239·8-639·1) | 365·8 (235·5-673·5) | 289·2 (262·1-469·7) | 0·63 | 365·8 (238·4-681·8) | 275·9 (262·1-415·3) | 0·41 |
| CXCL10, pg/mL | 7·3 (3·8-21·0) | 7·0 (4·2-21·3) | 8·3 (3·4-12·5) | 0·51 | 6·4 (3·7-21·1) | 9·2 (5·2-12·5) | 0·83 |
| CXCL-11, pg/mL | 5302·0 (3606·1-7391·6) | 5270·6 (3706·0-7544·3) | 5448·6 (3257·1-7316·4) | 0·94 | 5270·6 (3806·0-7696·9) | 5448·6 (3257·1-6733·0) | 0·77 |
| CCL-2, pg/mL | 55·5 (36·3-157·9) | 49·1 (36·3-132·8) | 77·2 (55·5-219·7) | 0·22 | 49·1 (36·1-141·2) | 77·2 (65·8-209·0) | 0·10 |
| CCL-3, pg/mL | 19·4 (11·6-36·5) | 18·9 (11·6-29·8) | 37·6 (14·0-79·7) | 0·080 | 18·9 (11·6-30·0) | 41·1 (19·4-79·7) | 0·077 |
| CCL-4, pg/mL | 64·2 (46·3-96·9) | 64·8 (46·6-90·0) | 55·4 (43·5-319·1) | 0·87 | 64·8 (46·3-91·3) | 55·4 (51·5-156·8) | 0·76 |
| CCL-5, pg/mL | 4864·5 (3607·0-8019·5) | 4727·8 (3540·0-7126·3) | 6507·0 (4059·0-10280·5) | 0·11 | 4856·5 (3607·0-7287·5) | 5462·5 (4059·0-9260·0) | 0·44 |

All data are presented as median (IQR), IQR = Interquartile range (25^th^ to 75^th^ percentile). After adjusting the comparison between progressors and non-progressors for baseline CD4+ T cell count for all biomarkers using logistic regression with a linear relationship between each biomarker and the logit, there was no change in the findings. The p-value for CCL-5 after adjusting for baseline CD4+ T cell count was 0·037 (Grade) and 0·095 (kPa).
