# Supplementary material for: Predictors of liver disease progression in people living with HIV-HBV co-infection on antiretroviral therapy
Source: eBioMedicine. 2024 Mar 21;102:105054. doi: 10.1016/j.ebiom.2024.105054 (PMC10966452; doi:10.1016/j.ebiom.2024.105054)
Supplement: Stastical analysis plan [file mmc2.pdf]

Statistical Analysis Plan

(SAP)

Study of Fibrosis and Immune Activation (SOFIA) – 3-year follow-up

Version: Final version 2

Authors: Dr Kasha Singh
Doherty Institute

Dr Jennifer Audsley
Doherty Institute

Sabine Braat (Statistician)
University of Melbourne

Professor Sharon Lewin
Doherty Institute

Version Number and Date	Summary of changes
1.0 dated 06/12/2017	Initial final version
2.0 dated 15/08/2022	Updated after final data collection (details Section 13)

Table of Contents

1 INTRODUCTION 3
2 DATA SOURCE 3
3 ANALYSIS OBJECTIVES 3
3.1 Aim 1 3
3.1 Aim 2 3
4 ANALYSIS SETS/ POPULATIONS/SUBGROUPS 4
5 ENDPOINTS AND COVARIATES 4
6 HANDLING OF MISSING VALUES AND OTHER DATA CONVENTIONS 5
7 STATISTICAL METHODOLOGY 5
7.1 STATISTICAL PROCEDURES 5
7.1.1 Aim 1 6
7.1.2 Aim 2 7
7.2 MEASURES TO ADJUST FOR MULTIPLICITY, CONFOUNDERS, HETEROGENEITY, ETC. 7
8 SENSITIVITY ANALYSES 7
9 SAMPLE SIZE 7
10 QC PLANS 8
11 PROGRAMMING PLANS 8
12 REFERENCES 8
13 CHANGES TO THE INITIAL FINAL VERSION 1 9
Appendix 1 - Schedule of assessments 10

1 INTRODUCTION

In order to determine what drives liver disease progression in HIV-HBV co-infected patients, a group of 67 patients on suppressive HBV-active HAART were prospectively followed and changes in liver disease progression were measured over 3 years, with study visits every 6 months (baseline, 6, 12, 18, 24, 30, and 36 months of follow-up) [1, 2]. The schedule of assessments is listed in Appendix 1. Liver fibrosis (assessed by transient elastography) and peripheral blood mononuclear cells (PBMCs) were assessed at baseline then annually.

Table 1 Participant recruitment by clinical site and overall

Site	Provided informed consent & follow-up post baseline visit	Follow-up at 3 years
HIV-NAT, Bangkok	31/67 (46.3%)	30/58 (51.7%)
Australia:	36/67 (53.7%)	24/58 (41.3%)
The Alfred - Melbourne	20/67 (31.3%)	14/58 (24.1%)
Melbourne Sexual Health Centre (MSHC)	10/67 (14.9%)	8/58 (13.8%)
St Vincent – Sydney (SVH)	6/67 (9.0%)	6/58 (10.3%)
Overall	67 (100%)	58/67 (86.6%)

2 DATA SOURCE

Data was collected using paper case report forms (CRFs), peripheral blood mononuclear cells (PBMC), plasma samples and Fibroscans (transient elastography). CRF data was entered into an Access database. Once all data was entered into Access, it was exported to Excel, checked and cleaned. Data clarifications were sent to sites as necessary, and any changes to the database resulting from clarification were logged in an electronic file. Samples collected were analysed at the Lewin/Cameron Laboratory, Doherty for markers of immune activation and microbial translocation. All data was de-identified and kept securely at the Doherty Institute on password-protected computers, files are backed-up and maintained by University of Melbourne IT.

3 ANALYSIS OBJECTIVES

3.1 Aim 1

To determine if changes in microbial translocation or inflammation can predict liver disease progression in HIV-HBV infected individuals receiving HBV-active HAART. We hypothesise that microbial translocation and markers of immune activation are associated with liver disease severity and liver disease progression in HIV-HBV co-infection.

3.1 Aim 2

To explore whether any of the key subgroups differ in fibrosis progression. More details on the defined subgroups of interest can be found in Section 4.

4 ANALYSIS SETS/ POPULATIONS/SUBGROUPS

The analysis set consists of all 67 participants across all sites. Liver disease progression as measured using Fibroscan is defined as an increase in Fibroscan Stage (F1≤5.8 kPa (kiloPascals), F2 5.9-7.5 kPa, F3 7.6-9.3 kPa, F4>9.4 kPa) from baseline result to last study time point (either 36 months or last completed data point). These patients are referred to as "**progressors**". Patients in the study are expected to show either no change, regression (decrease in Fibroscan Stage), or progression. Patients who do not show progression are referred to as "**non-progressors**". Some participants' fibrosis stage may fluctuate between first and last Fibroscan, such cases will be described but status will be defined only on first and last Fibroscan result. In addition, a pre-determined secondary definition of fibrosis progression was added, defined as 20% increase and ≥2 kPa (at least one value >5.9 kPa) [3].

Key subgroups of interest based on study entry characteristics include:

- HBeAg status at baseline (positive vs negative)
- Nadir CD4 cell count at baseline (>200 vs ≤200)
- CD4 nadir (%) (<10 or ≥10%)
- HIV viral load at CD4 nadir at baseline (>200,000 vs ≤ 200,000)
- ART duration (≤10 or >10 years)
- Derangement of ALT from normal range (yes vs no) – cut-offs are gender and site specific

Other exploratory subgroups (depending on numbers in each subgroup): gender (male vs female) and site (Bangkok vs others).

5 ENDPOINTS AND COVARIATES

Table 2 Endpoints and covariates

Grouping	Variable	Comment
Endpoints (measured repeatedly over time)		
Microbial translocation/ immune activation<br>Collected 6-monthly (7)<br>analysed yearly (4)	sCD14 (pg/mL)<br>CXCL10 (pg/mL)	Continuous<br>Continuous
Hepatic Inflammation<br>Collected 6-monthly (7)<br>analysed yearly (4)	ALT (IU/L)<br>AST (IU/L)<br>Albumin (g/L)	Continuous<br>Continuous<br>Continuous
Liver disease severity<br>Collected yearly (4)	Fibrosis (kPa)	Continuous
Liver disease progression<br>One value (1)	Progression (Grade change) (yes/no) (primary)<br>Progression (kPa change) (yes/no) (secondary)	Binary<br>Binary
Markers of cell death<br>Collected 6-monthly (7)<br>analysed yearly (4)	HMGB1 (ng/mL)	Continuous
Immune mediators<br>Collected 6-monthly (7)<br>analysed yearly (4)	TNF-α (pg/mL)<br>IL-10 (pg/mL)<br>IL-18 (pg/mL)<br>CXCL-9 (pg/mL)<br>CXCL-11 (pg/mL)<br>CCL-2 (pg/mL)	Continuous<br>Continuous<br>Continuous<br>Continuous<br>Continuous<br>Continuous

Grouping	Variable	Comment
	CCL-3 (pg/mL)<br>CCL-4 (pg/mL)<br>CCL-5 (pg/mL)	Continuous<br>Continuous<br>Continuous
CD4+<br>CD8+	CD4+ T-cell count (cells/μL)<br>CD4+ T-cells (%)<br>CD8+ T-cell count (cells/μL)<br>CD8+ T-cells (%)<br>CD4:CD8 ratio	Continuous<br>Continuous<br>Continuous<br>Continuous<br>Continuous
Covariates (measured once at study entry)		
Demographics	Gender (male/female*)<br>(* TransGender Female categorised as Female)<br>Age (years)<br>Body Mass Index (kg/m^2)<br>Site/Country	Binary<br><br>Continuous<br>Continuous<br>Binary
Adherence and alcohol questionnaires	Alcohol excess intake (yes/no)	Binary
CD4 nadir<br>CD4+<br>CD8+	Nadir CD4 cell count (cells/μL)<br>Nadir CD4 cell count (>200/≤200 cells/μL)<br>Nadir CD4 cells (%)<br>Nadir CD4 cells (<10/≥10%)<br>CD4+ T-cell count (cells/μL)<br>CD4+ T-cells (%)<br>CD8+ T-cell count (cells/μL)	Continuous<br>Binary<br>Continuous<br>Binary<br>Continuous<br>Continuous<br>Continuous
HIV RNA at time of CD4 nadir	HIV viral load at CD4 nadir (cps/mL)<br>HIV viral load at CD4 nadir (>200,000/≤200,000 cps/mL)	Continuous<br>Binary
Hepatitis serology	HBeAg status (positive/negative)<br>ALT (IU/mL)<br>ALT derangement from normal range (yes/no)	Binary<br>Continuous<br>Binary
HIV characteristics	Duration of ART (>10/≤10 years)<br>Time on ART (years)	Binary<br>Continuous
Treatment	HBV-active agents (<2/2)	Binary
Liver fibrosis	Fibroscan stage (1/2/3/4)<br>Fibroscan score (≤F2, >F2)	Categorical<br>Binary

6 HANDLING OF MISSING VALUES AND OTHER DATA CONVENTIONS

Summary statistics (e.g., percentages) will be based on non-missing values. When applying the mixed model regression model, the underlying assumption on missing data is missingness at most at random.

7 STATISTICAL METHODOLOGY

7.1 STATISTICAL PROCEDURES

A study flow-chart will be created to depict the flow of study participants over time based on the available data of the key outcome (i.e., liver disease).

Key baseline parameters will be summarised to describe the study cohort underlying all analyses. Using the variables presented in this table, we will explore whether progressors and non-progressors differ in any of the key variables at baseline.

We will test for differences between progressors and non-progressors using the wilcoxon rank-sum test for continuous data and the chi-square test or Fisher's exact test for categorical data. These key baseline parameters will also be summarised by subgroup (see Section 4) to explore for differences, using tests similar to those described for progressors/non-progressors. Continuous data will be presented as median with 25th - 75th percentile if data are considered skewed. Categorical data will be presented as frequency and percentages (based on non-missing data points).

7.1.1 Aim 1

Summaries will be presented of all endpoints (microbial translocation/immune activation outcomes, hepatic inflammation outcomes, and liver disease outcomes) listed in Table 2 at baseline and each follow-up time point and change from baseline to each follow-up time point. These summaries will also be created separately for those who are progressors or non-progressors. If the endpoint requires transformation (e.g., log transformation in case of positively skewed data) before fitting of a model (see below), summaries will also be presented on the transformed scale.

Longitudinal plots will be created of the endpoints, depicting more than one endpoint together in a graph to visually explore trends over time within and between endpoints, presented both by visit and change from baseline to each follow-up visit. These graphs will also be created separately for those who are progressors or non-progressors. If the endpoint requires transformation (e.g., log transformation in case of positively skewed data) before fitting of a model (see below), plots will also be presented on the transformed scale.

Correlations within and between endpoints at each visit and change from baseline visit will be explored (e.g., correlation sCD14 and Fibroscan score at month 12) and between consecutive follow-up visits and change from baseline visit separately (e.g., correlation sCD14 at month 12 with Fibroscan score at month 24). The Spearman correlation, exploring the degree to which the relationship between two outcomes is monotonic, will be used between two continuous outcomes and between an ordinal and continuous outcome, and point-biserial correlation between a binary and continuous outcome.

The following univariate models will be fitted to estimate the association between with the known risk factors (covariates) listed in Table 2 with each endpoint seperately, both univariable (one covariate - unadjusted) and multivariable (more than one covariate - adjusted):

1. Univariate logistic regression model with the binary outcome of liver disease progression during the study (yes/no) adjusted for CD4+ T-cells at study entry (except for variables related to CD4).
2. Univariate mixed regression model with each outcome the repeated continuous outcome in microbial translocation/immune activation, hepatic inflammation, and Fibroscan score (kPa) adjusted for CD4+ T-cells at study entry (except for variables related to CD4).

In model 2 the functional form of time in the model will be explored (i.e., linear or curvilinear [e.g., quadratic, cubic]), a random intercept will be included in these models to represent a random effect for subject and the structure of the residual (within-patient) variance-covariance matrix will explored (e.g., unstructured, AR(1),…).

We will examine longitudinal liver stiffness measured by Fibroscan as a continuous variable in kPA and compare between those classified as progressors and non-progressors via univariate mixed regression.

7.1.2 Aim 2

Longitudinal plots by subgroup will be created of the microbial translocation/immune activation outcomes, hepatic inflammation outcomes, and liver disease outcomes. We will start by exploring subgroups in the univariate models outlined in Aim 1 by including appropriate interaction terms. It should be noted that the sample size will be a limiting factor in detecting statistically significant subgroup differences.

Analyses will be conducted using STATA, SPSS, or SAS.

7.2 MEASURES TO ADJUST FOR MULTIPLICITY, CONFOUNDERS, HETEROGENEITY, ETC.

No adjustment for multiple testing is planned. In addition to p-values and 95% confidence intervals, associations are to be interpreted based on the magnitude and direction of the association.

8 SENSITIVITY ANALYSES

No sensitivity analyses are planned.

9 SAMPLE SIZE

Participants were recruited as part of a larger multi-centre cross sectional and longitudinal cohort which had multiple aims related to liver disease pathogenesis in HIV-HBV co-infection and HIV persistence in the liver. The sample size paragraph below relates to the larger cohort (n=100) that was planned in a previous grant

Planned sample size: Persons who experience liver disease progression during the follow-up period are expected to have an increase in liver stiffness (kPa) over time. Based on a recent retrospective study of liver stiffness using only 2 fibroscan measures in 92 HIV-HBV co-infected patients we expect that at least 8% of patients will have evidence of increased liver stiffness on HAART. Assuming the same distribution of LPS as described in grant APP 1024406, a sample of 100 patients will have 80% power at the 5% significance level to detect a minimum odds ratio of liver disease progression of 2.8 in patients with LPS in the upper quartile compared to the lower quartile.

Actual sample size: A sample of 67 patients will have less than 80% power at the 5% significance level to detect a minimum odds ratio of liver disease progression of 2.8 in patients with LPS in the upper quartile compared to the lower quartile.

10 QC PLANS

All data has been reviewed and cleaned. Data discrepancies have been resolved before start of the analysis and changes required to the raw data before the analysis have been documented in a file and applied to the raw data before start of the analysis.

The analysis output will be spot checked.

11 PROGRAMMING PLANS

No.	Tables & Listings
1	Demographic, clinical, fibrosis and immune activation characteristics at baseline<br>Overall and by subgroup
2A	Summary of alcohol questionnaire parameters over time<br>Overall and by subgroup
2B	Summary of full blood count parameters over time<br>Overall and by subgroup
2C	Summary of biochemistry and liver function profile parameters over time<br>Overall and by subgroup
2D	Summary of HBV RNA level parameters over time<br>Overall and by subgroup
2E	Summary of hepatitis serology parameters over time<br>Overall and by subgroup
2F	Summary of Fibroscan parameters over time<br>Overall and by subgroup
2G	Summary of study bloods parameters over time<br>Overall and by subgroup
3A1	Summary of microbial translocation/immune activation outcome parameters over time<br>by progressor status
3A2	Longitudinal plot of microbial translocation/immune activation outcome parameters over time -<br>by progressor status
3B1	Summary of hepatic inflammation outcome parameters over time<br>by progressor status
3B2	Longitudinal plot of hepatic inflammation outcome parameters over time<br>by progressor status
3C1	Summary of liver disease outcome parameters over time<br>by progressor status
3C2	Longitudinal plot of liver disease outcome parameters over time<br>by progressor status
4A	Correlation of microbial translocation/immune activation, hepatic inflammation, and liver disease outcome parameters (yearly)
4B	Correlation of microbial translocation/immune activation, hepatic inflammation, and liver disease outcome parameters (change from baseline to yearly)
5	Univariate modelling related to Aim 1
6	Subgroup explorations related to Aim 2

12 REFERENCES

1. Protocol (Version 7.0 dated 21 Jan 2016)
2. NHMRC project grant APP 1024406

3. Christiansen KM, Mossner BK, Hansen JF, Jarnbjer EF, Pedersen C, Christensen PB. Liver stiffness measurement among patients with chronic hepatitis B and C: results from a 5-year prospective study. PLoS One. 2014;9(11):e111912

13 CHANGES TO THE INITIAL FINAL VERSION 1

- Section 4 – Analysis Sets/Populations/Subgroups:

In addition to the primary definition of progressor, a pre-determined secondary definition of fibrosis progression was added, defined as 20% increase and ≥2 kPa (at least one value >5.9 kPa) as reported by Christiansen KM, Mossner BK, Hansen JF, Jarnbjer EF, Pedersen C, Christensen PB. Liver stiffness measurement among patients with chronic hepatitis B and C: results from a 5-year prospective study. PLoS One. 2014;9(11):e111912

The subgroup defined by derangement of AST from normal range was removed and the subgroups of CD4 nadir (%) (<10 or ≥10%) and ART duration (≤10 or >10 years) were added in order to manage low numbers (and group by meaningful cutoffs).

- Section 5 – Endpoints and Covariates:

Further markers of cell death (HMGB1) and immune mediators (tumour necrosis factor (TNF)-α, interleukin (IL)-10 and IL-18, chemokine (C-X-C motif) ligand (CXCL)-9, -10, -11, C-C motif chemokine ligand (CCL)2, CCL3, CCL4 and CCL5) were analysed. LPS was removed as an outcome. In addition, further covariates of interest (e.g., site) were examined.

- Section 7.1 – Statistical Procedures:

In addition to unadjusted models, adjusted models were fitted to account for differences in CD4+ T-cells at study entry (except for variables related to CD4). The analysis exploring correlation between two outcomes was removed due to the limitations to fit the proposed model in the data once available. As a result, no correlations or bivariate models were obtained. Statistical model 3 (mild vs severe Fibrosis over time) and model 4 (Fibrosis stage over time) were not fitted in favour of descriptively visualising the changes during the 3-year follow-up instead.

- Section 9 – Sample Size:

Participants were recruited as part of a larger multi-centre cross sectional and longitudinal cohort which had multiple aims related to liver disease pathogenesis in HIV-HBV co-infection and HIV persistence in the liver. The sample size paragraph included in the SAP previously relates to the larger cohort (n=100) that was planned in a previous grant.

- Section 10 – QC Plans:

In addition to the listed variables, the following variables were also derived to support post-hoc subgroup analyses (ART duration [≤10 or >10 years] and CD4 nadir (%) [<10 or ≥10%]) or description of the sample (HBV active agents [<2 or 2]).

Appendix 1 - Schedule of assessments

Assessment	Baseline visit (0)	Follow-up visits					
		1 (Month 6)	2 (Month 12)	3 (Month 18)	4 (Month 24)	5 (Month 30)	6 (Month 36)
Informed consent	X						
History	X						
Clinical examination	X	X	X	X	X	X	X
Concurrent medications & conditions	X	X	X	X	X	X	X
Adherence & alcohol questionnaires	X	X	X	X	X	X	X
Full Blood Count (FBC)	X	X	X	X	X	X	X
Biochemistry and liver function profile	X	X	X	X	X	X	X
HIV RNA level	X	X	X	X	X	X	X
CD4/CD8	X	X	X	X	X	X	X
Hepatitis serology:							
HBsAg	X	X	X	X	X	X	X
Anti-HBs	X	X	X	X	X	X	X
HBeAg	X	X	X	X	X	X	X
Anti-HBe	X	X	X	X	X	X	X
Anti-HB core	X						
Delta Ab (exclusion)	X						
Anti-HCV (exclusion	X						
HBV DNA level	X	X	X	X	X	X	X
Fibroscan	X		X		X		X
Study bloods:							
Plasma storage	X	X	X	X	X	X	X
PBMCs	X		X		X		X
